# An NFκB-dependent mechanism of tumor cell plasticity and lateral transmission of aggressive features

**DOI:** 10.18632/oncotarget.25465

**Published:** 2018-06-01

**Authors:** Nancy Adriana Espinoza-Sánchez, Jennifer Enciso, Rosana Pelayo, Ezequiel M. Fuentes-Pananá

**Affiliations:** ^1^ Programa de Doctorado en Ciencias Biomédicas, Facultad de Medicina, Universidad Nacional Autónoma de México, C.P. 04510, Ciudad de México, México; ^2^ Programa de Posgrado en Ciencias Bioquímicas, Universidad Nacional Autónoma de México, C.P. 04510, Ciudad de México, México; ^3^ Centro de Ciencias de la Complejidad, Universidad Nacional Autónoma de México, C.P. 04510, Ciudad de México, México; ^4^ Centro de Investigación Biomédica de Oriente, Instituto Mexicano del Seguro Social, C.P. 74360, Metepec, Puebla, México; ^5^ Unidad de Investigación en Virología y Cáncer, Hospital Infantil de México Federico Gómez, C.P. 06720, Ciudad de México, México

**Keywords:** Intra-tumor heterogeneity and intra-clonal communication, tumor aggression, cancer stem cells, invasion, NFκB and STAT signaling pathways

## Abstract

Breast cancer is a complex disease exhibiting extensive inter- and intra-tumor heterogeneity. Inflammation is a well-known driver of cancer progression, often attributed to immune cells infiltrating the tumor stroma. However, tumor cells themselves are capable to secrete a variety of inflammatory molecules, of which we understand very little about their role in intra-clonal communication. We recently reported the capacity of triple negative cell lines to induce a cancer stem cell (CSC)-like phenotype and invasion properties into luminal cells, a mechanism mediated by pro-inflammatory cytokines that up-regulated the CXCL12/CXCR4/CXCR7 chemokine signaling axis. We performed transcriptional array analyses of CSCs-associated genes and cancer-inflammatory cell crosstalk genes and built regulatory networks with the data collected. We found a specific molecular signature segregating with the induced-invasive/stemness phenotype. Regulatory network analysis pointed out to an NFκB transcriptional signature, active in aggressive triple negative cells and in induced-invasive/CSC-like luminal cells. In agreement, NFκB inhibition abolished the induction of the stemness/invasive features. These data support an NFκB dependent mechanism of intra-clonal communication responsible for tumor cell plasticity leading the acquisition of cancer aggressive features. Understanding the communication between different tumor clones would help to find better therapeutic and prophylactic targets to prevent BrC progression and relapse.

## INTRODUCTION

Breast cancer (BrC) is the leading cause of cancer mortality in working age women. Although prevalence is higher in developed countries, mortality is greater in developing countries in which most cases are diagnosed at advanced stages [1, 2]. BrC is a highly heterogeneous disease from which we have identified different molecular subtypes that are used to predict the clinical outcome. Expression of estrogen receptor (ER), progesterone receptor (PR) and the human epidermal growth factor receptor (HER2/ERBB2/CD340) is extensively used to classify BrC into luminal, HER2 positive and triple negative subtypes. In-depth analysis of gene expression signatures has helped to understand the origin of the different disease subtypes [3–5].

More recently, we have also recognized intra-tumor heterogeneity as an important factor contributing to disease aggression [6]. Intra-tumor heterogeneity is deeply influenced by the composition of the tumor stroma, which is formed by the extracellular matrix (ECM), multiple types of immune cells, connective tissue cells, blood, and lymph vessels [7]. Bi-directional communication between tumor cells and stromal inflammatory cells is a critical driver of cancer initiation and progression [8]. The link between chronic inflammation and carcinogenesis was first proposed by Rudolf Virchow in 1863 based on the consistent presence of leukocytes in the stroma of tumors [9]. Now, mounting evidence supports the capacity of tumor cells to harness immune cells to fulfill pro-tumoral functions, through secreted cytokines, chemokines, growth factors, and other bioactive compounds [7, 8, 10].

Another layer of intra-tumor heterogeneity is given by the co-existence of genetically/epigenetically distinct tumor clones. Tumor intra-clonal cooperation has also been documented, in particular between metastatic and non-metastatic clones [11–13]. This intra-clonal communication not only helps tumor cell invasion, but it is also proposed to favor chemoresistance and disease relapse [14]. We have recently reported that co-culturing triple negative and luminal BrC cells leads to lateral transmission of aggressive features, characterized by luminal cells acquiring a cancer stem cell (CSC)-like phenotype and invasion properties [15]. Indeed, co-existence of different BrC molecular subtypes within the same tumor has been reported, for instance, ER positive and ER negative or HER2 positive and HER2 negative clones [16]. Also, BrCs of a clinical good prognosis at presentation sometimes exhibit intrinsic chemoresistance or relapse as an aggressive variant [6].

Tumor heterogeneity and intra-clonal cooperation could be explained by a high degree of cell plasticity. At the center of tumor cell plasticity lies the epithelial to mesenchymal transition (EMT), which often accounts for the capacity of terminally differentiated tumor cells to detach from the tumor, become mobile and invasive, migrate to distant organs and establish secondary tumor growths [17]. EMT- and CSC- associated-features are often observed together, and metastatic BrC cells often share characteristics of both processes [18–20]. Surprisingly, although TGF-β is arguable the main molecule responsible for EMT [21], we could not find evidence of TGF-β driving the lateral transmission of aggressive features [15]. Instead, pro-inflammatory cytokines G-CSF, GM-CSF, IL-8 and MCP-1 seemed to be up-regulating the CXCL12/CXCR4/CXCR7 chemokine signaling axis and explaining the induced-invasive/stemness phenotype observed in our previous study [15].

Communication between tumor cells and immune cells present in the tumor stroma has been extensively studied resulting in novel therapeutic drugs developed against immune checkpoint controls [22, 23]. Interestingly, we observed that the set of inflammatory cytokines driving intra-clonal cooperation is highly similar to the one that directs communication between cancer cells and monocytes/macrophages, also with tumor promoting consequences [24]. However, how secreted inflammatory molecules mediate communication between different tumor clones and how this mechanism of tumor autocrine communication contributes to BrC intra-clonal heterogeneity, disease progression and prognosis is an issue significantly less explored.

In this study, to better understand the potential mechanism of tumor intra-clonal communication, we performed transcriptional array analyses of CSCs-associated genes and cancer-inflammatory cell crosstalk genes, finding specific signatures segregating with an induced-invasive/stemness phenotype. Regulatory network analysis pointed out to NFκB, STAT3, and AR (Androgen Receptor) pathways as important drivers of the transmission of aggressive features. In agreement, an NFκB intrinsic transcriptional signature was observed in aggressive triple negative cell lines and in induced- invasive/CSC-like luminal cells. Furthermore, NFκB inhibition abolished the lateral transmission of aggressive features, supporting the importance of this transcription factor for tumor cell plasticity and intra-clonal communication. Understanding the mechanisms promoting intra-clonal heterogeneity and communication would help us to find better therapeutic and prophylactic targets to prevent BrC progression and relapse.

## RESULTS

### Cancer stem cell genes associated with the induced-invasive/stemness phenotype

We previously reported that highly aggressive triple negative cell lines (HA-BrC: HS578T and MDA-MB-231) produced high concentrations of pro-inflammatory cytokines G-CSF, GM-CSF, IL-8, and MCP-1, and the conditioned media (CM) of these cells induced aggressive features in non-aggressive luminal cell lines (NA-BrC: MCF-7 and T47D). This lateral transmission of aggressive features consisted of NA-BrC cells losing the EMT marker E-cadherin, gaining stemness markers CD44, OCT-4 and SOX-2, and acquiring the capacity to form large spheres in low-adherent plates. Furthermore, induced luminal cells upregulated the CXCL12 receptors CXCR4 and CXCR7 becoming invasive in response to CXCL12. We called this process the inducible-invasive/CSC-like phenotype [15]. A CSC-associated gene expression array was performed to identify the genes differentially regulated during this induced stage, see Supplementary Figure 1 for a schematic representation of the experimental design.

In our previous study [15], we found intrinsic differences to both aggressive-induced NA-BrC cells with respect to a panel of up-regulated chemokine receptors, chemoattraction to FBS and frequency of positive cells to CSC markers. Here, we also observed an intrinsic transcriptional signature particular to each cell line, which may explain those differential responses. The average raw data obtained with the array is shown in Supplementary Table 1. A supervised Student's *t*-test performed only on MCF-7 cells identified *PLAT, EGF*, *ENG, PLAUR, DLL1* and *KITLG* genes with a *p*-value < 0.05. *PLAT*, *EGF, ENG, PLAUR* and *DLL1* were up-regulated, while *KITLG* was down-regulated (Supplementary Figure 2A). T47D cells showed 18 genes with a *p*-value < 0.05: *CD44, NF*κ*B1, ALDH1A1, STAT3*, *NANOG, SAV1, JAG1, PROM1, DDR1, ALCAM, GSK3*β, *PLAT, GAPDH, FGFR2, WEE1, CHEK1, LIN28A,* and *ID1*; the first twelve genes showed increased expression, while the last six decreased upon exposure to HA-CMs (Supplementary Figure 2B). An analysis based on the non-induced (basal) gene expression profile of MCF-7 and T47D cell lines supports inherent differences between both cell lines (Supplementary Figure 2C). Only four elements of this group of basally expressed genes overlap with the set of genes differentially regulated upon treatment with the HA-BrC cells CMs (*DLL1* in MCF-7 and *JAG1, DDR1*, and *FGFR2* in T47D cells).

**Figure 1:**
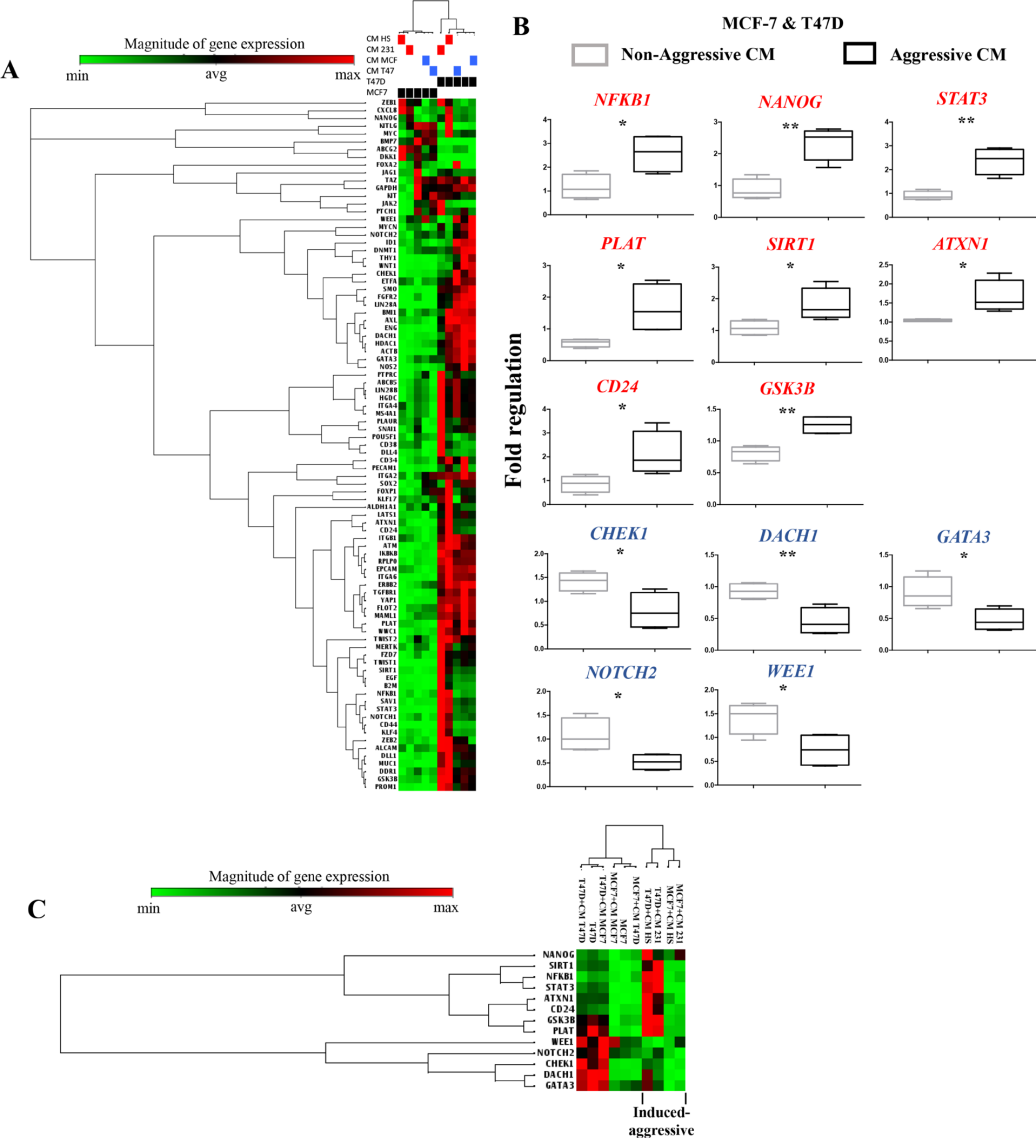
Gene expression signature associated with cancer stem cells during the acquisition of the induced invasive/CSC-like phenotype (**A**) Unsupervised hierarchical clustering and heat map of the CSC-array genes expression after MCF-7 and T47D cells (black boxes) were cultured with their regular media, their own NA-CM (blue boxes) or the CM from the HA-BrC cells (red boxes). (**B**) Supervised analysis using the Student's *t*-test of genes differentially expressed in NA-BrC cells after culturing with the NA- and HA- conditioned media. Genes shown in red were up-regulated and those in blue were down-regulated. (**C**) Unsupervised hierarchical clustering and heat map based on the genes found after the statistical analysis in B. Two independent biological replicates were analyzed.

A one-way unsupervised hierarchical clustering analysis supported that both NA-BrC cells significantly changed CSC-gene expression in response to both HA-BrC stimuli, with both NA-BrC cells showing a distinctive profile that separated them from cells treated with NA-CMs and from unstimulated cells (Figure 1A). Thirteen genes were identified separating induced-aggressive and non-induced cells with significant differences (*p*-value < 0.05), of which eight were up-regulated in NA-BrC cells after HA-BrC stimuli: *NF*κ*B1*, *NANOG*, *STAT3, PLAT*, *SIRT1*, *ATXN1*, *CD24* and *GSK3*β, while five were down-regulated: *CHEK1*, *DACH1*, *GATA3*, *NOTCH2*, and *WEE1* (Figure 1B and Supplementary Table 2). Although expressed at different levels in induced-MCF-7 and -T47D cells, a hierarchical unsupervised clustering of these genes allowed a clearer separation between cells cultivated with NA-CMs from those cultivated with HA-CMs (Figure 1C). This group of genes potentially represents an overlap between the mechanisms by which MCF-7 and T47D cells acquire the induced-invasive/CSC-like phenotype. A Cancer Stem Cell Transcription Factor Activation Array (Signosis, Inc, number FA-1004) also found KLF4, MYC, NANOG, OCT-3/4, SOX-2 and SNAIL activated in both MCF-7 and T47D after treatment with the CM of HA-BrC cells (data not shown). Altogether, these data support that particular signatures of CSC-related gene expression mark the acquisition of the induced-invasive/CSC-like phenotype with some elements common to both MCF-7 and T47D cells.

### Identifying signaling pathways responsible for the induced-invasive/CSC-like phenotype

**Figure 2:**
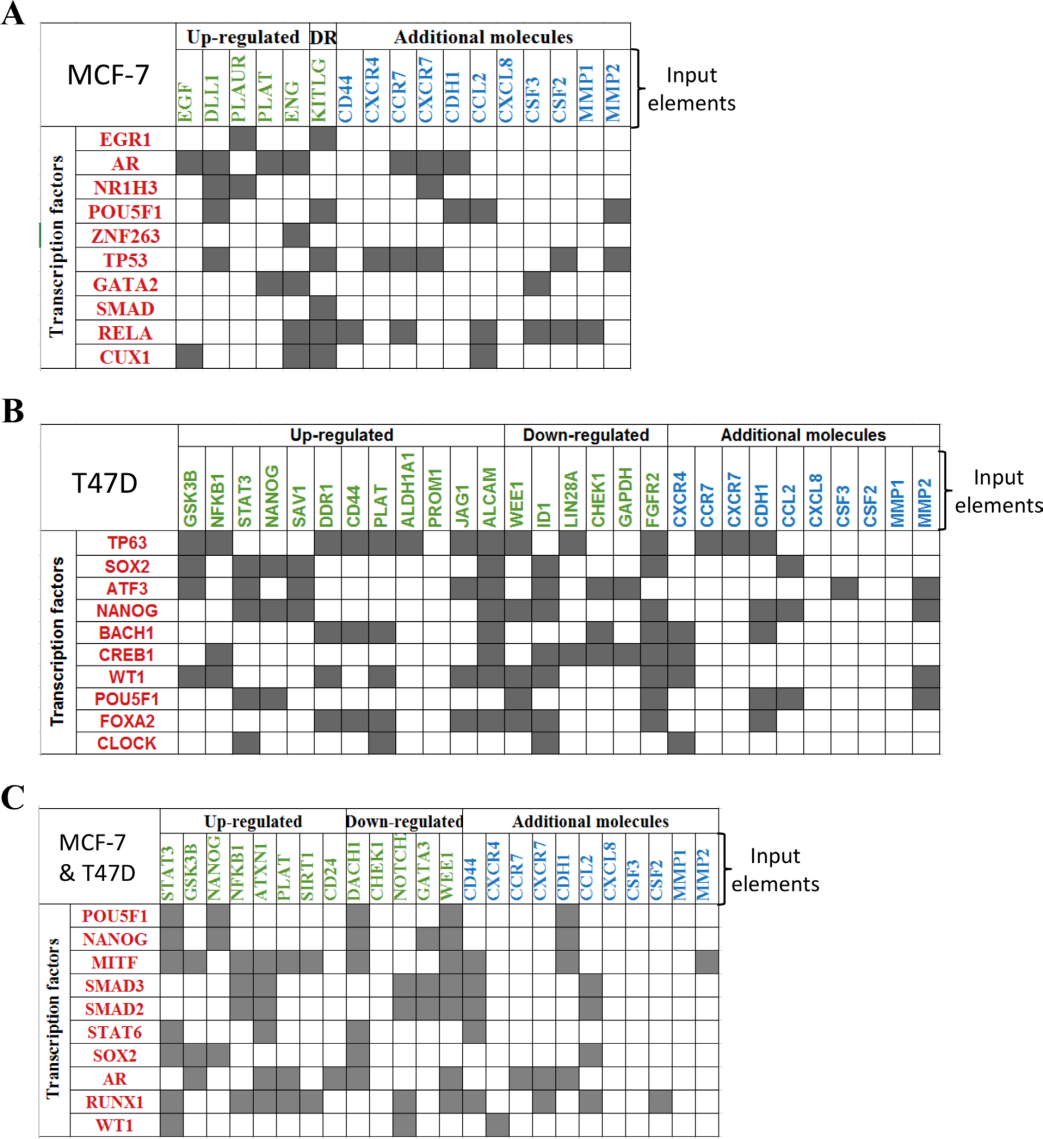
ChiP-X enrichment analysis of ChEA database infers transcription factors regulating the induced invasive/stemness phenotype Transcription factors (TF) inferred during this analysis are shown in red. These TFs are potential upstream regulators of the genes found with the CSC gene expression array (shown in green) and other molecules that we previously found experimentally, shown as additional molecules in blue [15]. This analysis was performed individually to MCF-7 cells (**A**), T47D cells (**B**) and for the genes observed with the jointed analysis of both cell lines (**C**).

In order to identify the transcriptional signals driving the differentially expressed genes in the induced-invasive/CSC-like BrC cells, we performed a ChiP-X transcription factor enrichment analysis followed by the construction of functional interaction networks. For this analysis, in addition to the gene array data, we used the molecules that we previously observed that were differentially regulated in induced-MCF-7 and -T47D cells, individually or the ones shared by both cell lines (these group of genes consisted of CD44, CDH1, CXCR4, CXCR7, CCR7, G-CSF/CSF3, GM-CSF/CSF2, IL-8/CXCL8, MCP-1/CCL2 and metalloproteinases (MMP) -1 and -2, and they were taken from reference [15]). These datasets of input elements suggested the participation of EGR1, AR (androgen receptor), NR1H3, POU5F1 (OCT-4), ZNF263, TP53, GATA2, SMAD, RELA, and CUX1 in MCF-7 cells; and of TP63, SOX2, ATF3, NANOG, BACH1, CREB1, WT1, POU5F1, FOXA2 and CLOCK in T47D cells (Figure 2A and 2B). When the analysis was performed using as input dataset the elements common to both cell lines (those found in Figure 1C and reference 15), ten transcription factors were retrieved: POU5F1, NANOG, MITF, SMAD3, SMAD2, STAT6, SOX2, AR, RUNX1 and WT1 (Figure 2C and Supplementary Table 3). In agreement with our previous experimental data, SOX-2 and POUF5F1/OCT-4 appeared as important regulatory transcription factors [15].

**Figure 3:**
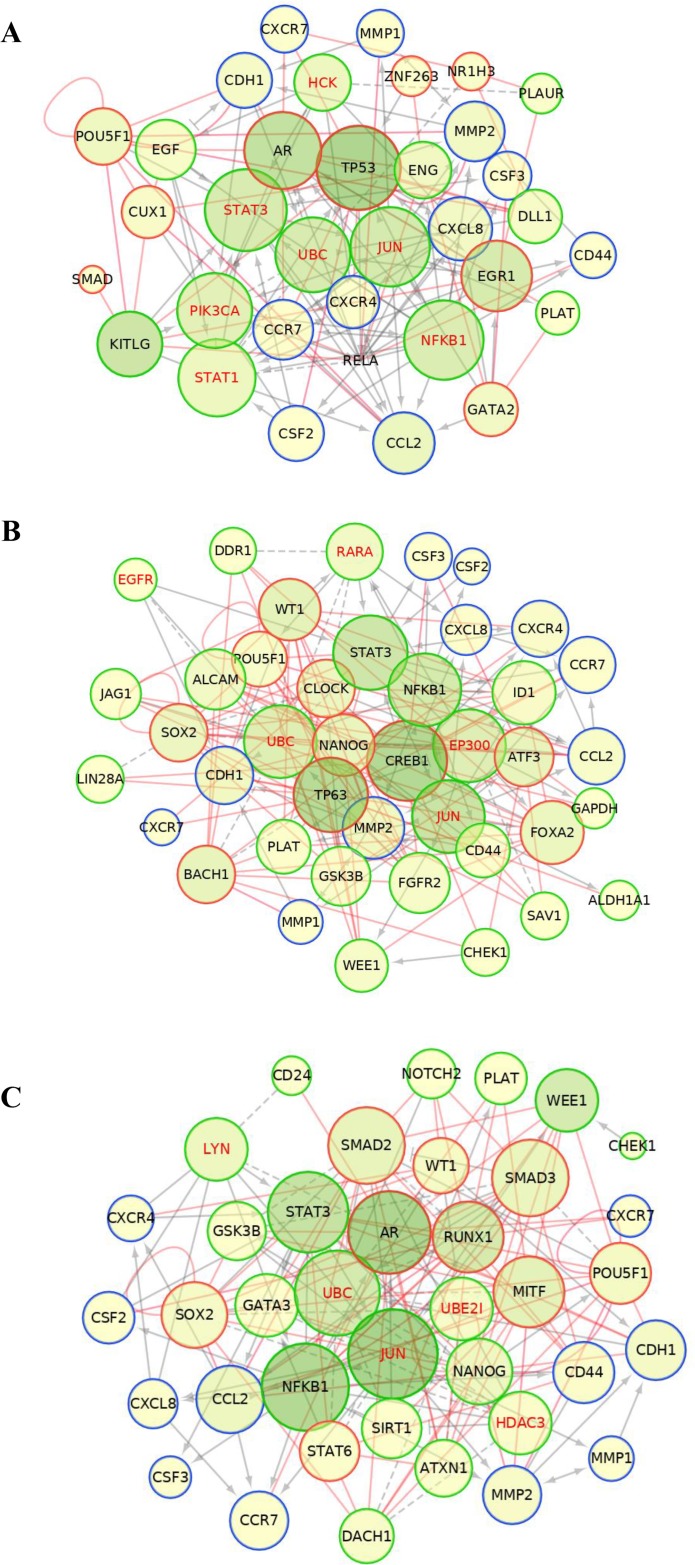
Protein-protein interaction networks Construction of the functional interaction (FI) networks for MCF-7 (**A**), T47D (**B**) and the jointed analysis of both cell lines (**C**), inferred using a list of input genes that included: the differentially expressed genes identified in the CSC array (green nodes), the potentially inferred transcriptional factors (red nodes) and the set of molecules found experimentally in reference [15] (blue nodes). Grey solid and dashed lines represent protein-protein interactions obtained from Reactome plug-in Cytoscape, and red lines represent the transcriptional regulations inferred from the TF enrichment analysis. Influence of each node was addressed through node betweenness and closeness centrality represented by the color intensity and size of the node, respectively. Nodes with greater influence are represented with larger radius and darker green.

We then constructed functional interaction (FI) networks using the set of input genes that included: the differentially expressed genes identified with the CSC array, the Chip-X inferred transcription factors and the set of molecules found experimentally in each induced NA-BrC cell line, individually or the ones common to both cell lines. The FI network was generated with the Reactome FI plugin for Cytoscape [25, 26] that accesses the Reactome manually curated FI network constructed from pathway databases. The FI network helps to find functional interactions within a defined set of genes. The influence of the nodes in the FI network was then estimated by calculating the closeness centrality and betweenness centrality scores. The closeness centrality determines how close a node is to all other nodes in a network, and it is calculated by the inverse of the sum of the shortest distance between the analyzed node and every other node in the network. The betweenness centrality indicates the control of a node over information passing through the network, and its calculation is based on the number of shortest paths that pass through the referred node. The Reactome FI suggested five elements that connect most of the input genes in a single network shared in both induced BrC cell lines: JUN, UBC, STAT3, and NFKB1, while TP53, PI3K, and EGR1 seem to be particular of MCF-7 cells, and CREB1, TP63, EP300 of T47D cells (Figure 3A and 3B). In agreement, when we analyzed both induced cells as a group with the set of data shared by both NA-BrC cells, we also observed STAT3, AR, UBC, JUN, and NFKB1 as the central pathways connecting most elements of the network. Molecules involved in ubiquitination (UBC and UB2I), HDAC3 (Histone Deacetylase 3) and c-Jun (JUN) interconnect the negatively regulated genes (*CHEK1*, *DACH1*, *GATA3*, *NOTCH2* and *WEE1*), of which c-Jun, a transcription factor downstream of MAP kinases, seems to be the most important [27]. On the other hand, the chemokine receptors and cytokines nodes seem to be strongly regulated mainly through NFκB1, but also through STAT3 and AR (Figure 3C). Particularly, the NFκB pathway seems to be central to the induction of the aggressive phenotype as it is suggested by the separated and joined FI networks of both induced invasive/CSC-like MCF-7 and T47D cells. We assessed the basal expression of *NFKB1*, *IKBKB*, *JAK2* and *STAT3* in the array data of HS578T and MDA-MB-231 cells observing that both HA-BrC cell lines exhibit an elevated basal expression of *IKBKB* and *NFKB1*, compared with the basal level of expression of MCF-7 and T47D cells (Supplementary Figure 3A). A similar observation, although less striking, was made for the STAT3 pathway, finding *JAK2* elevated in HS578T cells and less in MDA-MB-231, and *STAT3* elevated just in MDA-MB-231 cells (Supplementary Figure 3B).

**Figure 4:**
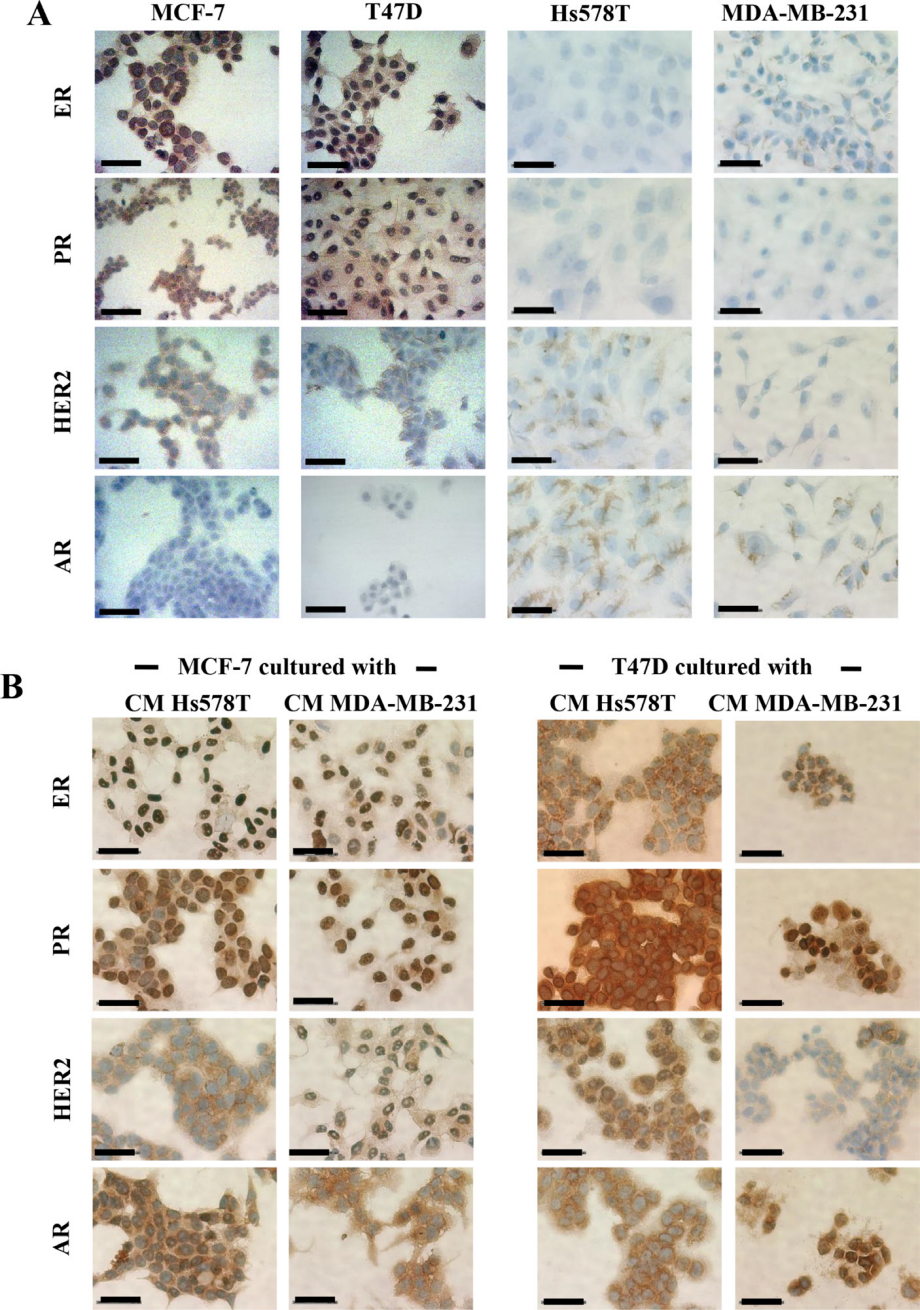
Induced invasive/CSC-like cells do not alter their luminal phenotype but turn on expression of AR Immunohistochemistry of ER, PR, HER2, and AR expression in (**A**) all BrC cell lines under basal conditions and (**B**) MCF-7 and T47D cells cultured with CM from HA-BrC cell lines. Positive cells are brown based on DAB chromogen. Nuclei were stained with hematoxylin (blue). The scale bars indicate 50 μm. Magnification 400×.

We then addressed whether induced-invasive/CSC-like MCF-7 and T47D cells changed their molecular phenotype as they acquire aggressive features. We examined ER (Estrogen Receptor), PR (Progesterone Receptor) and HER-2 expression by immunocytochemistry in all cell lines, confirming the luminal phenotype of the NA-BrC cell lines MCF-7 and T47D (positive to ER, PR and only weakly positive to HER2), and the triple negative phenotype of HA-BrC cell lines (Figure 4A). Hs578T showed a few cells positive to HER2. Because of the data supporting AR expression, we also assessed the expression of this receptor observing that the luminal cells were negative, while the triple negative cells were positive. In positive cells, ER, PR and AR had nuclear expression while HER2 had membrane expression. When the NA-BrC cells were cultured with the HA-CMs they did not change the expression of ER, PR and HER2. Interestingly, we observed that AR expression was induced by the effect of the HA-CMs (Figure 4B). These observations corroborated the activation of AR suggested by the FI networks of Figure 3.

### The NFκB pathway at the center of the lateral transmission of aggressive features

**Figure 5:**
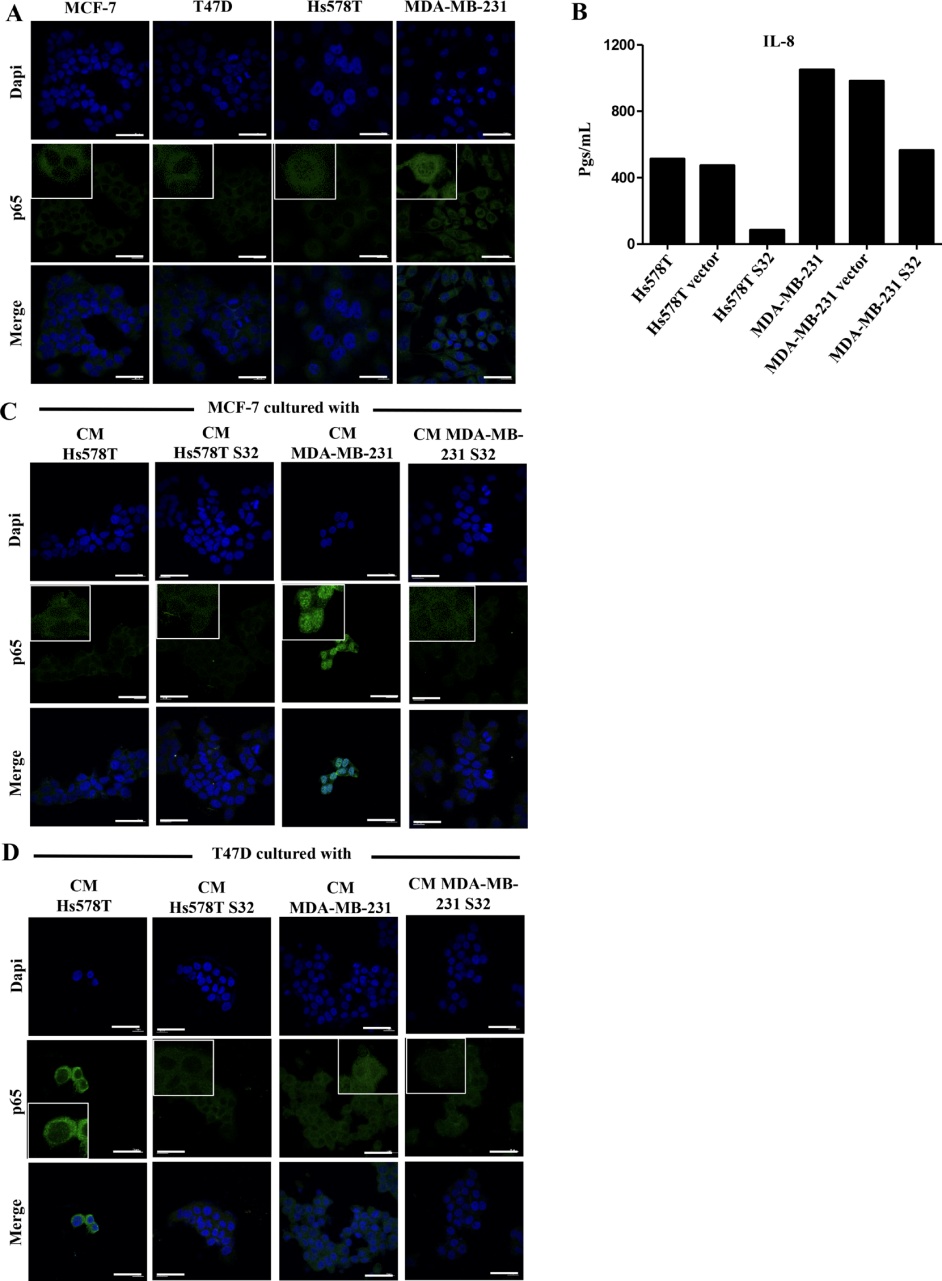
p65 is present in the nucleus of HA-BrC cells and its translocation to nucleus is induced in invasive/CSC-like NA-BrC cells (**A**) Analysis of the constitutive cellular localization of p65 in NA- and HA-BrC cell lines by immunofluorescence (IF). (**B**) The concentration of IL-8 (in pgs/mL) was measured in the CM from the HA-BrC cell lines that were transiently transfected with a dominant negative IκB (HS578T S32 and MDA-MB-231 S32), and controls in which the cells were not transfected or transfected with an empty vector. p65 expression by IF in (**C**) MCF-7 and (**D**) T47D cells that were cultured with CM from HA-BrC cell lines HS578T S32 and MDA-MB-231 S32, and controls in which NFκB was not inhibited. The scale bars indicate 20 μm. Magnification 600× with inserts further amplifying the original image. Representative images are shown. Two independent biological replicates were analyzed and representative images were chosen.

Our data support a model in which the NFkB signaling pathway is required for the aggressive behavior of HS578T and MDA-MB-231 cells, and that the lateral transmission of aggressive features also depends on activation of this pathway in the induced cells. We tested whether the p65 subunit of NFκB was present in cytoplasm or nucleus of the HA-BrC and NA-BrC cells, finding that p65 is cytoplasmic in the NA-BrC cell lines while it is mostly nuclear in the HA-BrC cell lines (Figure 5A). We performed a transient transfection assay of HA-BrC cells Hs578T and MDA-MB-231 with a dominant-negative IκB (mutated in serine-32 and serine-36) to inactivate the canonical NFκB pathway. Since IL-8 transcription is regulated by NFκB [28], we tested the concentration of IL-8 in the CM of inhibited and non-inhibited cells by an ELISA test. We found that the inhibition of the NFκB pathway reduces the secretion of IL-8 in both HA-BrC cell lines (Figure 5B). We observed a better p65 nuclear signal in MDA-MB-231 cells than in Hs578T cells, which also correlated with increased secretion of IL-8. We then subjected both NA-BrC cells to CMs of NFκB-active or -inactivated HA-BrC cells and assessed the cellular localization of p65. In MCF-7 cells, we observed nuclear p65 when it was cultivated with the CM of MDA-MB-231, whereas with the CM of the NFkB inhibited cells p65 was not translocated (Figure 5C); a similar but somehow fainter effect was observed with the Hs578T CM. Similarly, nuclear p65 was observed in T47D cells stimulated with both HA-CMs and mostly cytoplasmic p65 when the NFκB pathway was inhibited (Figure 5D).

**Figure 6:**
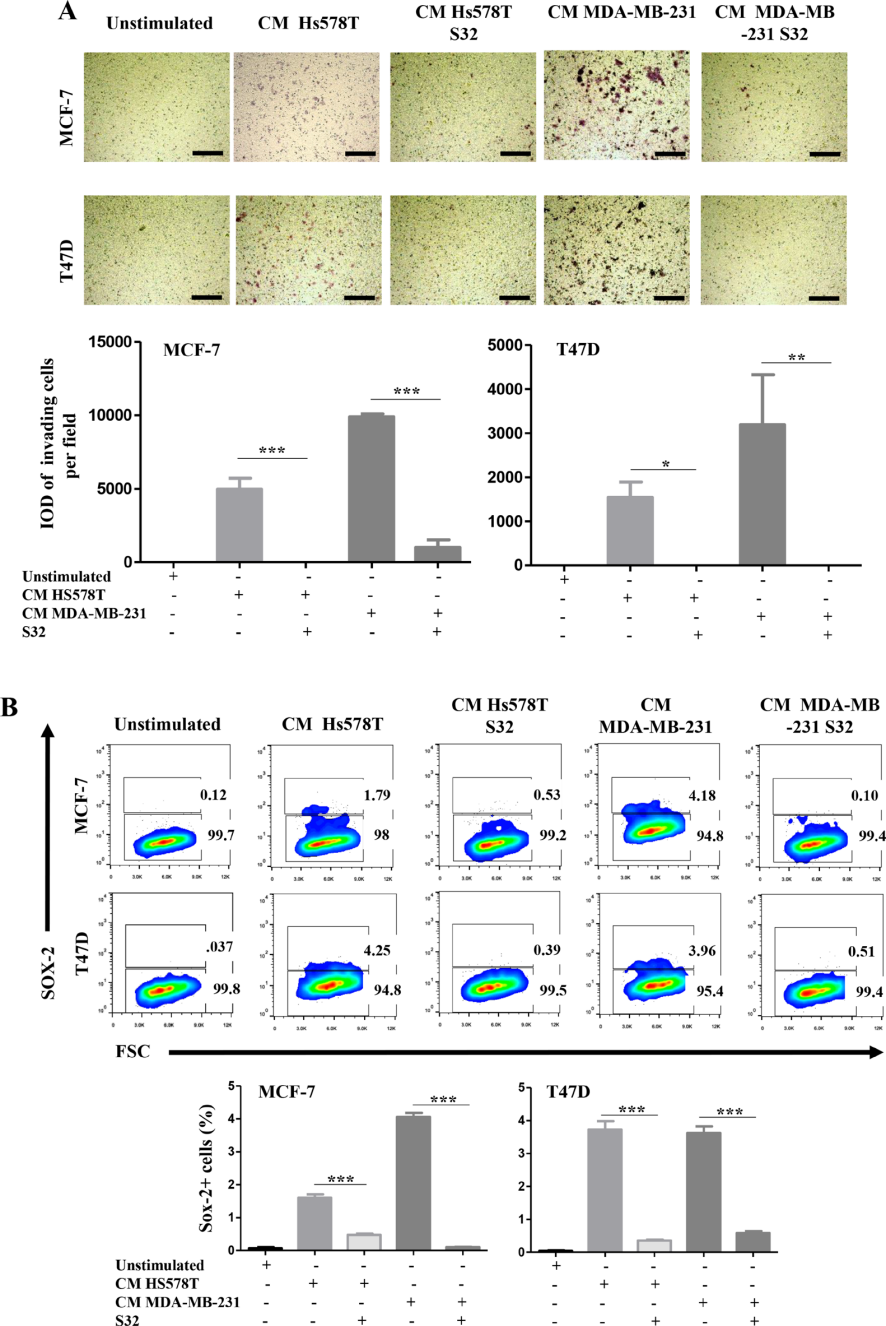
The induced-invasive/stemness phenotype depends on p65 translocation MCF-7 and T47D cells were cultured with the CM from HA-BrC cell lines in which the cells were transiently transfected with the dominant negative IκB (HS578T S32 and MDA-MB-231 S32), and controls in which NFκB was not inhibited. After 72 hours of culture (**A**) transwell invasion assays were performed and (**B**) SOX-2 expression was measured by FACS. Upper panels show representative images of invasion assays and cell density plots, while the frequency of invading cells and SOX-2 positive cells are graphed below. The data represent the mean ± SEM (Standard Error of the Mean) of 3 independent experiments, representative images are shown. ^*^P < 0.05 and ^***^P < 0.001. The scale bars indicate 100 μm. Magnification 100×.

We then performed invasion assays and measured the frequency of SOX-2 positive cells in NA-BrC cells induced with HA-BrC CMs in which the NFκB pathway was inhibited by the transient expression of the dominant negative mutant IκB. Figure 6A shows that MCF-7 cells completely abolished their invasive capacity when they were cultured with the CM from the NFkB-inhibited HS578T, whereas with the CM of the NFkB-inhibited MDA-MB-231 cells, the invasion was also significantly reduced [average integrated optical density (IOD) = 9,800 per field *vs* IOD = 1,000 per field]. Likewise, T47D cells were not invasive after treatment with both NFkB-inhibited CMs (Figure 6A). We also observed that the frequency of SOX-2 positive cells in both induced NA-BrC cell lines was reduced by the effect of the inhibition of the NFκB pathway (Figure 6B). Therefore, these data support that the ability of aggressive tumor cells to laterally transmit aggressive features to non-aggressive cells depends on the basal activity of the NFkB pathway.

**Figure 7:**
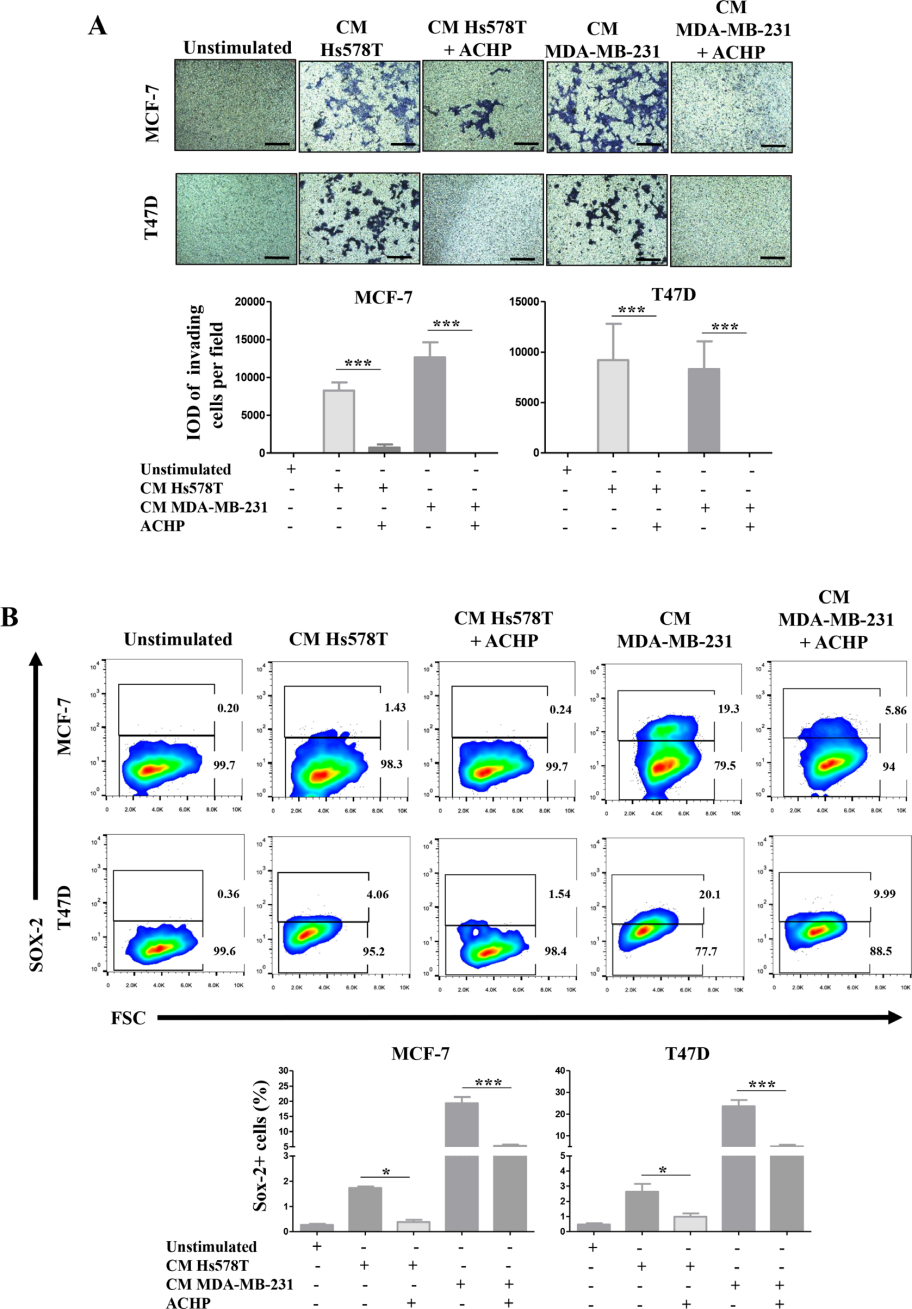
Pharmacological inhibition of NFκB activity blocks the induced-invasive/stemness phenotype MCF-7 and T47D cells were cultured with CM from the HA-BrC cell lines in which the NFκB pathway was inhibited with ACHP or were treated with DMSO as vehicle control. After 72 hours of culture (**A**) transwell invasion assays were performed and (**B**) SOX-2 expression was measured by FACS. Upper panels show representative images of invasion assays and cell density plots, while the frequency of invading cells and SOX-2 positive cells are graphed below. The data represent the mean ± SEM of 3 independent experiments. ^*^P < 0.05 and ^***^P < 0.001. The scale bars indicate 100 μm. Magnification 100×.

We also drug-inhibited NFkB with ACHP in Hs578T and MDA-MB-231 cells and treated the NA-BrC cells with the CMs of inhibited cultures. To test whether the induced invasive/stemness phenotype is affected, we subjected the NA-BrC cells to an invasion assay and measured the frequency of SOX-2 positive cells. Figure 7A shows that MCF-7 cells almost completely reduced their invasive capacity when they were cultured with the CM from the NFkB-inhibited HS578T cells [average integrated optical density (IOD) = 8,200 per field *vs* IOD = 719 per field], whereas with the CM of the NFkB-inhibited MDA-MB-231 cells invasion was completely abolished. Likewise, T47D cells were not invasive after treatment with both CMs from NFkB-inhibited HA-BrC cells. When we evaluated the expression of SOX-2 by flow cytometry we also observed a significant decrease of SOX-2 positive cells in comparison with the NA-BrC cells stimulated with HA-CMs without the inhibition of the NFκB pathway (Figure 7B).

### A survival and immunomodulatory signature is also part of the induced-aggressive features in primary breast cancer cells

**Figure 8:**
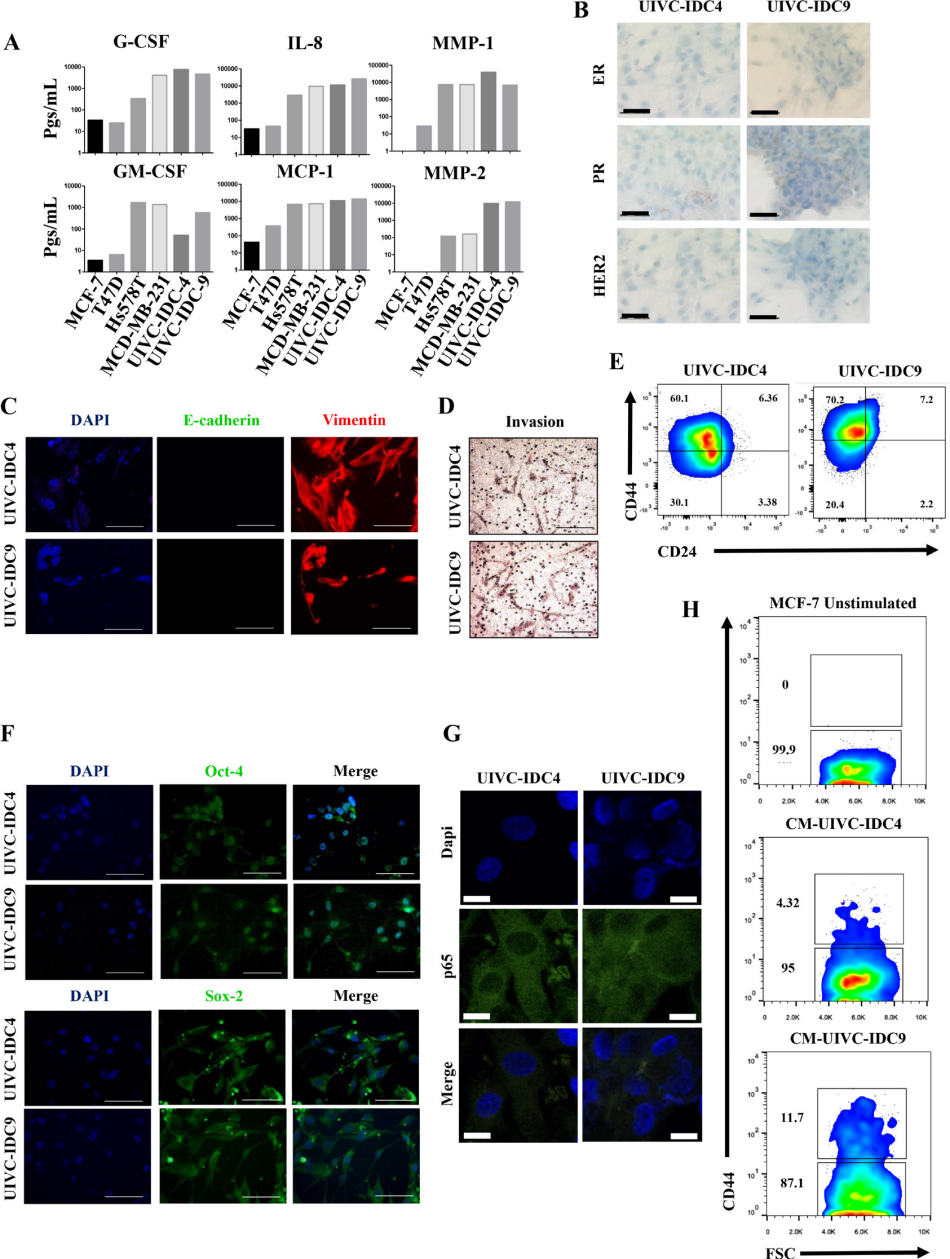
Primary breast cancer cultures also induced the invasive/stemness phenotype (**A**) Milliplex assays were performed to determine the basal concentration of pro-inflammatory mediators and metalloproteinases (expressed in pgs/ml) in the CMs of NA-, HA-BrC cells and two primary BrC cultures. The following analyses were carried out in the primary BrC cultures: (**B**) expression of hormonal receptors were measured by immunocytochemistry (ICQ), (**C**) the epithelial to mesenchymal transition was assessed by Immunofluorescence (IF), (**D**) invasiveness in transwell assays, (**E**) expression of the stemness markers CD44 and CD24 was measured by flow cytometry, (**F**) expression of the stemness markers OCT-4 and SOX-2 was measured by IF, and (**G**) p65 cellular localization was measured by IF. (**H**) MCF-7 cells were cultured with the CM from both primary cell lines and CD44 was examined by flow cytometry. The data represent the mean ± SEM of 3 independent experiments, representative images are shown. The scale bars indicate 20 μm in IF images and 50 μm in ICQ images. IF images magnification of 600× and ICQ images of 400×.

Because the capacity of lateral transmission of aggressive features is highly correlative with an inflammatory profile, we also tested an array that measures the expression of genes related to cancer and inflammation cross-talk. For this, we wanted to explore breast cancer primary cultures that although considered non-aggressive because of their clinical staging and histological grading, they were highly inflammatory in culture, particularly of the inflammatory molecules that we have previously reported for Hs578T and MDA-MB-231 cells: G-SCF, IL-8, MMP1, GM-CSF, MCP-1 and MMP2. We have previously reported the isolation of eight primary cultures from Mexican BrC patients [24]. Two of those cultures, UIVC-IDC4 and UIVC-IDC9, even though they were classified as clinical stage IIA and IIB, respectively, and histological grade 2 exhibited a similar inflammatory signature to that of Hs578T and MDA-MB-231 cells (Figure 8A). Both primary cultures are in an early passage, and when we tested expression of ER, PR and HER2 to the cultured cells they were triple negative, with only a very few cells staining for PR (Figure 8B). They also exhibited an EMT-like phenotype with expression of vimentin and absence of E-cadherin (Figure 8C) and were invasive in transwell assays (Figure 8D). In both primary cultures, the EMT correlated with a CSC-like phenotype characterized by CD44 positive and CD24 low to negative expression (Figure 8E), and also positivity to the stemness associated transcription factors OCT-4 and SOX-2 (Figure 8F). We assessed whether the NFkB pathway was activated in both primary cultures and by immunofluorescence observed an intense staining of p65, which was present in both cytoplasm and nucleus (Figure 8G). Finally, we corroborated that the CM of these primary cultures induced the expression of CD44 on MCF-7 cells, as a sign of their capacity to induce the aggressive invasive/CSC-like phenotype (Figure 8H).

**Figure 9:**
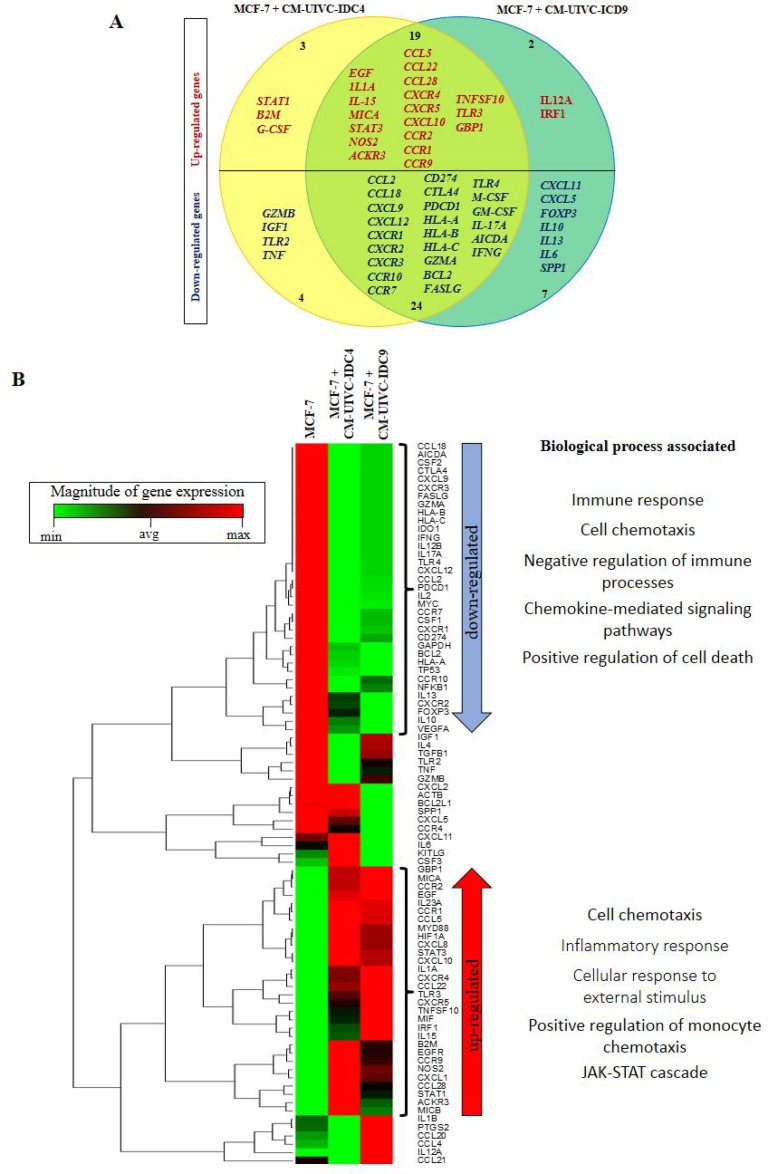
Expression of cancer-inflammation crosstalk genes is induced by primary breast cancer cells MCF-7 cells were cultured with the CM of the primary BrC cells and expression of genes associated with Cancer Inflammation and Immunity Crosstalk was analyzed by a PCR array. (**A**) Venn diagram indicating the overlap of expression of genes that were up- (red) or down-regulated (blue) in MCF-7 cells treated with the CM of UIVC-IDC4 (yellow) or UIVC-IDC9 (dark green) cells. (**B**) Unsupervised hierarchical clustering analysis and heat map according to expression patterns, and biological processes associated with the genes that were up- or down-regulated. Red and green in the heatmap diagram represent higher and lower expression, compared with the median for each particular gene. One PCR array was performed.

To obtain a complete picture of the inflammatory profile of induced invasive/CSC-like BrC cells, we then analyzed the expression of a panel of cancer and inflammation cross-talk genes in MCF-7 cells exposed to the CMs of the BrC primary cultures. We found that MCF-7 cells stimulated with both CMs shared 19 up-regulated and 24 down-regulated genes (Figure 9A). The average raw data obtained with the array is shown in Supplementary Table 4. Of note, we observed again up-regulation of *STAT3*, *EGF*, *NOS2*, *CXCR4*, *CXCR5*, *CCL5* (RANTES) and *G-CSF* as we have found early on in this study and in our previous reports [15, 24]. One-way unsupervised hierarchical clustering analysis showed how MCF-7 cells responded to the CMs clustering away from un-induced cells (Figure 9B). Also, the heat map unveiled the genes whose differential expression allowed induced cells to cluster together. We used the STRING bioinformatic tool to find biological processes associated with those genes. Because this is an expression array of inflammation-related genes, we found processes linked to immune responses and chemotaxis in both down- and up-regulated genes. Still, an inflammatory signature better correlated with up-regulated genes, together with the JAK/STAT signaling pathway, while a signature denoting resistance to cell death was found among down-regulated genes. Particularly important is a signature of down-regulated immunomodulatory genes, which are often found controlling anti-tumoral immunosurveillance responses (*GZMA*, *GZMB*, *TLR2*, *TLR4*, *HLA-A*, -*B*, -*C*, *CTLA4*, *PDCD1*, *CD274*, *FASLG*, *FOXP3*, *IL-10*, *IFNG* and *SPP1*) (see Supplementary Tables 5 and 6 for all processes found). Down-regulation of this set of genes is a common feature of aggressive cancers.

## DISCUSSION

BrC is a very complex disease due to its great heterogeneity, both inter- and intra-tumoral. The histological classification of BrC into molecular subtypes has helped to give more targeted therapies. However, an increasingly recognized problem is that different tumor cells and/or different tumor regions often express different molecular markers [29, 30]. The normal breast is *per se* composed of functionally different types of cells, and Van Keymeulen *et al.* demonstrated in an animal model that breast stem cells contribute to the development of a cellular diverse fully-functional mammary gland [31]. These multipotent properties of stem cells, together with the high plasticity of tumor cells could favor the co-existence of more than one molecular subtype within the same tumor. Studies in mice showed that oncogenic mutations in *PIK3CA* or *BRCA1* switch the phenotype of luminal cells to basal cells with stemness properties [32, 33]. Jordan *et al.* described that in patients with ER+/HER2− primary breast tumors, their circulating tumor cells (CTC) were HER2+. Interestingly, HER2+ and HER2- populations exhibited different activation levels of multiple signaling pathways and were also differentially sensitive to chemotherapy, and when both populations were individually expanded, they originated daughter cells with the reverse phenotype [16]. Likewise, Yates *et al.* using whole genome and targeted sequencing approaches found a diversity of molecular subtypes in different areas of tumors in patients with invasive BrC [34]. Recently, Cejalvo and collaborators working with 123 paired primary and metastatic tissues found evidence of subtype conversion, mainly from luminal A to luminal B, but also from luminal to HER2 tumors [35]. Also supporting tumor cell heterogeneity, Ithimakin and collaborators observed that luminal tumors presented a subpopulation of HER2+ ALDH+ (Aldehyde dehydrogenase) CSC-like cells at the tumor invasive front. Administration of the anti-HER2 antibody trastuzumab as prophylactic therapy abolished tumor growth in mice xenotransplanted with luminal HER2 negative BrC cells [36]. This group also observed that expression of HER2 depended on Receptor Activator of NFκB (RANK)-ligand in luminal cells infiltrating the bone marrow and that trastuzumab reduced the expression of HER2 and ALDH in these metastatic cells [36]. Trastuzumab is already in phase II clinical trial to target HER2 negative BrCs [37]. BrC intra-clonal subtype co-existence and its clinical implications have recently and thoroughly been reviewed by Yeo *et al.* [38].

These studies support cancer inherent mechanisms of cell plasticity and of intra-tumor heterogeneity. However, we still lack plenty of our understanding of the complex interactions that exist between different tumor populations, the role of inflammation in this communication and how it influences BrC progression and prognosis. One of the first groups that demonstrated communication between different clones was Miller *et al.*, who observed cooperation between metastatic and non-metastatic tumor populations [12]. Using a syngeneic mouse model for small cell lung cancer, Calbo *et al.* observed that tumors are composed of different cells with neuroendocrine or mesenchymal phenotype, and the communication between them influenced the metastatic behavior of the neuroendocrine cells [11]. Marusyk *et al.* suggested that less represented tumor clones could influence other subpopulations to adapt to microenvironmental cues favoring the emergence of clones with novel phenotypes and functions [39]. Mukherjee *et al.*, showed that non-migratory cells induced a CXCR4-dependent metastatic potential into more differentiated cancer cells [13], similar to our previously published observations [15]. Cleary *et al.* observed the coexistence of Wnt1-expressing luminal cells and basal cells carrying an *Hras* mutation, and both clones were necessary to establish new tumors. When they targeted Wnt1 clones to promote tumor regression, the basal cells restored tumor growth by recruiting heterologous Wnt-expressing cells [40].

In this study, the analysis of stemness-related genes allowed us to obtain a wider picture of signaling pathways and transcription factors that may be responsible for the induction of an invasive/CSC-like phenotype. Functional interaction networks pointed out to NFκB as an important regulator of the lateral transmission of aggressive features and of tumor cell plasticity. Since the pioneer studies of Lehmann *et al.* [41], triple negative BrC (TNBC) cells have been described as a mix of different subtypes with genetic, transcriptional, histological and clinical differences, and with some clones exhibiting an immunomodulatory capacity based on the constitutive activation of the NFκB and JAK/STAT pathways [41]. Kuo and collaborators showed that a suicide gene therapy targeting the STAT3/ NFκB pathways in a TNBC cell line resulted in a reduction of tumor growth, loss of invasiveness and an enhanced sensitization of the TNBC cell line to cisplatin therapy [42]. Also, overexpression of the NFκB pathway-related transcription factor SP1 was associated with poor prognosis in doxorubicin-treated TNBC patients [43], and McDaniel *et al.*, demonstrated that activation of STAT3 is necessary for invasion and metastasis of TNBC cells [44]. Chua *et al.* found in MCF-10A cells that activation of the p65 NFκB subunit induced overexpression of ZEB1 and ZEB2, both master regulators of the EMT and that this also correlated with decreased expression of the epithelial marker E-cadherin and increased expression of the mesenchymal marker vimentin [45]. In a different study, TNBC cell invasiveness was reduced upon siRNA-mediated inhibition of the NFκB pathway [46]. Altogether, these and other studies [47, 48] support the participation of the NFκB and STAT3 pathways in the induction of an EMT- and stem cell-like phenotype that contributes to the tumor aggressive features.

NFκB is the most important driver of cytokine expression in immune cells. Inflammatory BrC (IBC) is one of the most aggressive subtypes of BrC, and overexpression of NFκB is associated with the IBC poor prognosis [49]. The activity of the NFκB pathway explains both the inflammatory profile and the high metastatic potential that characterizes this type of BrC. Also, a selective inhibitor peptide of the IκB kinase abolished the proliferative and survival capacity of an ER− HER2+ BrC cell line. In the same study, nuclear expression of p65 was detected in stromal cells of ER− HER2− tumors [50]. The authors speculated that activation of NFκB in stromal cells contributed to the communication between stroma and cancer cells and that this is probably mediated by cytokines. Indeed, MCP-1 and IL-8 are transcriptional targets of NFKB1 and RELA in tumor cells [51, 52]. We observed *PLAT* upregulation in both induced invasive/CSC-like NA-BrC cells, and PLAT is a well-known regulator of MMPs via the NFκB pathway [53]. We also observed STAT3 and AR as influential nodes of the interaction networks. STAT3 is also an important regulator of immune cell function, which has been previously linked to cancer [54]. Some studies support an NFκB and STAT3 crosstalk required for communication between tumor cells and their microenvironment (reviewed in [55]). Chung *et al.* reported that STAT3 activates hTERT (human telomerase reverse transcriptase) thought NFκB, which in turn was responsible for the increased expression of CD44 and the invasive and migratory capacity of BrC cells [56]. AR is one of the most important factors driving progression of prostate cancer [57, 58], but its role in breast cancer is less clear. Molecular subtyping of triple negative BrCs found an AR-specific signature [59]. Hu *et al.* found that close to 90% of BrCs are AR positive and, while AR+ luminal BrCs were of better prognosis, triple negative tumors were not influenced by the expression of AR [60]. On the contrary, Sutton *et al.* observed that AR+ triple negative BrCs have a reduced risk of recurrence and metastasis [61]. Interestingly, in prostate cancer, the JAK/STAT3 signaling pathway regulates expression of AR and this correlates with decreased survival [62].

We also observed a signature of deregulated immunomodulatory genes in induced-aggressive luminal cells, which are often found controlling anti-tumor immunosurveillance responses. This would argue that despite the fact of an initial diagnosis of good prognosis, the influence of the microenvironment and the tumor crosstalk could change the course of the disease. The understanding of the importance of anti-cancer T cell responses and immune checkpoint has been crucial for the development of novel cancer therapies. Among the genes that we found down-regulated are: *GZMA*, *GZMB*, *HLA-A*, -*B*, -*C* and *IFNG*, all supporting a model of impaired CD8 T cell cytotoxic activities, one of the most important anti-cancer responses [63]. Development of novel strategies targeting checkpoint proteins is currently changing the landscape of anti-cancer therapy. In 2015 the U. S. Food and Drug Administration (FDA) approved ipilimumab, a monoclonal antibody (mAb) that blocks CTLA-4, to treat unresectable or metastatic melanoma (https://www.cancer.gov/about-cancer/treatment/drugs/fda-ipilimumab). Currently, CTLA-4 mAbs are been tested in phase I-III trials in a variety of tumor types [23]. Likewise, the FDA has already approved the use of Pembrolizumab and Nivolumab, targeting PD-1/PD-L1 interaction, to treat a variety of human cancers [22, 23, 64].

In summary, this study illustrates the capacity of communication between different populations of BrC cells and how this contributes to the emergence of new clones with novel functions. We observed the NFκB pathway as a key regulator of this cell plasticity. Previous studies support the importance of the NFκB pathway in the aggressive features of TNBCs. We extend those studies supporting that TNBC cells with an intrinsic imprint of NFκB activity are able to induce an inflammatory profile that laterally activates the pathway, transmitting an aggressive capacity into less aggressive tumor clones. It is also worth mentioning that our bioinformatic analysis also points out to STAT3, AR, and JUN as potential collaborators of NFκB and that we also observed a strong signature of down-regulated immunomodulatory genes associated with the induced-aggressive cancer cells (see Supplementary Figure 4 for a depiction of a working model). The clinical importance of tumor cell plasticity relies on its contribution to tumor cell heterogeneity, which may explain why neoplasias considered of good prognosis at diagnosis sometimes relapse as aggressive and/or chemoresistant variants. The use of single-cell technologies would aid to better understand the nature of each tumor subpopulation and to guide the use of combinations of different primary and prophylactic therapies to improve the patient´s clinical outcome.

## MATERIALS AND METHODS

### Ethics statement

This study was approved by the Scientific, Ethical and Biosafety review boards of our institution (Comité de Investigación, Comité de Ética en Investigación and Comité de Bioseguridad of Hospital Infantil de México “Federico Gómez”).

### Cell culture

All cancer cell lines are mammary epithelial cells derived from metastatic sites except for Hs578T derived from the primary tumor site; all lines were obtained from the American Type Culture Collection (ATCC). Based on hormonal and growth factor receptor expression MCF-7 and T47D are classified as Luminal A (non-aggressive breast cancer cells, NA-BrC), and Hs578T and MDA-MB-231 as triple negative (high aggressive breast cancer cells, HA-BrC). MCF-7 (ATCC reference number HTB-22), T47D (HTB-133), Hs578T (HTB-126) and MDA-MB-231 (CRM-HTB-26) were cultured as described previously in [15]. Primary BrC cell lines UIVC-IDC4 and UIVC-IDC9 were obtained from the tissues and sera bank of the Unidad de Investigación en Virología y Cáncer, Hospital Infantil de México “Federico Gómez” (HIMFG). The characteristics of the patients and isolation of the primary tumor cells were described in [24]. Primary tumor cell isolates were cultured in DMEM/F12 supplemented with 5% horse serum, 100 U/mL penicillin and 100 μg/mL streptomycin, 100 ng/mL cholera toxin, 0.5 μg/mL hydrocortisone, 10 μg/mL insulin, and 20 ng/mL of EGF at 37° C in 5% CO^2^ atmosphere. Both primary isolates are under passage 10.

### Conditioned media from breast cancer cells

To obtain conditioned media 2 × 10^6^ cells were plated in 182 cm^2^ flasks in their standard supplemented medium. Supernatants were discarded when cultures reached 80% of confluence, cells were rinsed with PBS 1× (Phosphate Buffered Saline, GIBCO, REF 20012–027), and then 30 mL of culture media without horse serum and supplements were added. Conditioned media were harvested after incubation for an additional 48 hours, centrifuged at 1500 rpm/5 minutes, aliquoted, and stored at −20° C until use.

### qRT-PCR arrays

84 CSC related genes were analyzed using the Human Cancer Stem Cells RT^2^ Profiler PCR Array (REF PAHS-176Z) on induced invasive/stemness cells and controls (uninduced luminal and TNBC cells). The induction of the invasive/stemness phenotype was described in [15]. 84 Cancer Inflammation and Immunity Crosstalk related genes were also measured using the Human RT^2^ Profiler PCR Array (REF PAHS-181Z) in MCF-7 cultivated with the conditioned media from the two primary BrC cell cultures. Total RNA was extracted from 1 × 10^6^ cells using the RNeasy^®^ Mini Kit (REF 74104). The purity and quality of isolated RNA were determined by measuring the ratio of absorbance values at 260 and 280 nm. cDNA was synthesized from 1 μg of total RNA using the RT^2^ First Strand Kit (REF 330401), which includes the additional removal of genomic DNA from the sample and a specific control for reverse-transcription. qRT-PCR was performed using the RT^2^ Profiler PCR Array and a Master Mix RT^2^ SYBR Green ROX FAST Master Mix (REF 330623). Samples were run on a Rotor-Gene Q (Qiagen). All procedures were performed according to the manufacturer's instructions (all reagents are from Qiagen). The ∆∆CT value of each gene was normalized to the ‘household’ genes included in the array. Genes with expression changes of more than 2-fold after normalization were considered significant. Data Analysis was performed in the Qiagen Web portal available at pcrdataanalysis.sabiosciences.com. Two independent biological replicates were analyzed.

### Heatmap and clustergram analyses of gene expression data

The samples and conditions analyzed were as follows: MCF-7 and T47D cells were cultured with their regular media (conditions: unstimulated MCF-7 and T47D), or with their own CM or the CM from the HA-BrC cells (conditions: MCF-7 + CM MCF7, MCF-7 + CM T47D; T47D + CM MCF7, T47D + CM T47D; MCF-7 + CM HS, MCF-7 + CM 231; T47D + CM HS, T47D + CM 231). In the case of MCF-7 cultured with the conditioned media of two primary BrC cultures, the conditions analyzed were MCF-7 unstimulated (MCF-7) or MCF-7 + CM UIVC-IDC4 and MCF-7 + CM UIVC-IDC9. An unsupervised hierarchical clustergram and heatmap were constructed using the online RT^2^ Profiler PCR Array Data Analysis Web portal (Qiagen). Expression of the 84 CSC or 84 Cancer Inflammation and Immunity Crosstalk related genes were represented as a heatmap plot and samples were clustered according to their gene expression patterns.

### Statistical and bioinformatic analysis, and network representation

In order to identify the genes from the CSC array that were differentially expressed between NA-BrC cell lines cultured in NA- and HA- conditioned media or genes from the Cancer Inflammation and Immunity Crosstalk array that were differentially expressed between MCF-7 cells cultured with the conditioned media from the two primary BrC cell lines, fold-expression data were analyzed by an unpaired two-tailed *t*-test and considered statistically significant when a *p*-value < 0.05 was obtained. The *T*-test was performed with the R package ‘stats’, software version 3.3.3 [65]. The list of differentially expressed genes was fed into the transcription factor inference module of the search engine X2K version 1.6 (http://www.maayanlab.net/X2K/) to identify elements that were likely upstream regulators. The transcription factor inference was run using ChEA database that contains information from transcription factor ChIP-X studies (CHIP-chip, CHIP-seq, CHIP-PET) extracted from published data [66]. The background organism was set to “human”. A new gene list was generated including the differentially expressed genes, the top 10 transcriptional factors sorted by *p*-value and the genes that we reported previously involved in the induction of the aggressive invasive/stemness phenotype [15]. A functional interaction (FI) network was generated using the new gene list as an input of the Gene Set Analysis tool from the Reactome FI plugin [25] implemented in Cytoscape. The FI network was merged with the transcriptional interactions of ChEA analysis to obtain a complete network and analyze centrality measures (e.g. closeness centrality and betweenness centrality). Visualization and centrality measurements were done with the Cytoscape software version 3.5 [26]. We searched for the biological processes associated with the target genes identified that were up- or down-regulated in MCF-7 cells after they were cultured with the CM of primary isolates. The online platform STRING (https://string-db.org) was used for this purpose; this platform uses three different databases: GO (Gene Ontology), Pfam (Protein families) and KEGG (Kyoto Encyclopedia of Genes and Genomes).

### Inhibition of the NFκB pathway on HA-BrC cells

To inhibit the NFκB pathway in HA-BrC cells HS578T and MDA-MB-231, we used the selective IKKα and IKKβ inhibitor ACHP from Tocris Bio-techne brand (REF 4547) [67]. ACHP was prepared as a 20 mM stock solution in dimethyl sulfoxide (DMSO) and stored at −70° C until use. 2 × 10^6^ cells of HS578T and MDA-MB-231 were plated in 182 cm^2^ flasks in their standard supplemented medium, supernatants were discarded 24 hours later and cells were rinsed with PBS 1×, adding 30 mL of their respective culture media without FBS plus 16 nM/mL of ACHP. After 48 hours of culture, CMs were harvested, centrifuged at 1500 rpm/5 minutes, aliquoted, and stored at −20° C until use. As controls, we obtained CMs from cells cultures without ACHP. In order to generate a transient defective signal from the canonical NFkB pathway, we also transfected Hs578T and MDA-MB-231 cells with a plasmid vector expressing a dominant-negative IκB (mutated at serine 32 and 36) [68]. 24 hours after transfection, cells were washed with PBS 1×, after which we added their respective culture media without FBS. After 48 hours of culture, CMs were harvested, centrifuged at 1500 rpm/5 minutes, aliquoted, and stored at −20° C until use. MCF-7 and T47D cells were cultured with 3 mL of CM of HA-BrC cells cultured with ACHP or 3 mL of CM of HA-BrC cells transfected with the dominant negative IκB mutant. After incubation for 72 hours, MCF-7 and T47D cells were harvested and the induced invasive/stemness phenotype was evaluated.

### Invasion assay

Invasion assays were carried out with 2 × 10^4^ NA-BrC after the induction of the invasive/stemness phenotype using 10% of FBS as a chemoattractant, as reported in [15]. After 24 hours of incubation at 37° C, invasive cells were stained with crystal violet and observed using a microscope Motic AE31 and images were acquired with a digital camera (Moticam 5.0 MP). Crystal violet staining intensity was quantified using the Image-Pro Plus software, and the integrated optical density (IOD) of invading cells was reported.

### Flow cytometry

3 × 10^5^ NA-BrC cells were subjected to the induction of the invasive/stemness phenotype, after which cells were collected and stained to observe the expression of SOX-2 (mouse monoclonal anti-human SOX-2-Alexa 488 Clone: 245610, BD Biosciences, REF 560301), CD44 (mouse monoclonal anti-human CD44-PE, 1:50, Clone: G44-26, BD Biosciences, REF 555479) and CD24 (mouse monoclonal anti-human CD24-PECy7 1:50, Clone: ML5, BD Biosciences, REF 561646). The staining was done as described in [15]. Briefly, for extracellular staining, cells were blocked with an unspecific IgG antibody and then incubated with the primary antibodies. Cells were then incubated with 7AAD to exclude dead cells. For intracellular staining, cells were blocked with an unspecific IgG antibody, then were washed with Phosflow Perm/Wash Buffer I 1X (PPWB), and fixed and permeabilized using Cytofix/Cytoperm solution. To block intracellular Fc receptors the cells were again incubated with the unspecific IgG antibody diluted in PPWB, and then incubated with mouse monoclonal anti-human SOX-2. All acquisitions were performed on a Guava Easycyte 8 cytometer (Merck Millipore). Analysis of flow cytometry data was performed on viable 7-AAD negative cells (except for SOX-2 staining) using the FlowJo V10 software (TreeStar Inc).

### Immunofluorescence

3 × 10^4^ cells were seeded on coverslips for 24 hours after which, an immunofluorescence staining was performed as described in [15]. Briefly, cells were fixed with paraformaldehyde and permeabilized with Triton X-100. Cells were blocked and then stained overnight at 4° C with the primary antibody rabbit anti-human NFκB p65 antibody (1:500, Santa Cruz Biotechnology, REF sc-372) and the secondary antibody donkey anti-rabbit FITC (1:500, Jackson ImmunoResearch, REF 711-095-152). Also, UIVC-IDC4 and UIVC-IDC9 were stained with anti-E-Cadherin (1:100, Clone: 36/E-cadherin, BD Biosciences, REF 610181), rabbit monoclonal anti-vimentin-Alexa Fluor-594 (1:1000, Clone: EPR3776, REF ab154207), rabbit polyclonal anti-Oct4 (1:100, REF ab18976) or rabbit polyclonal anti-Sox2 (1:100, REF ab97959); all antibodies were from Abcam. The secondary antibodies: goat anti-mouse IgG-FITC (1:500 Sigma Aldrich Co., REF F0257) or the anti-rabbit mentioned above. Finally, nuclei were stained with DAPI. Images for p65 were acquired on a Nikon Ti Eclipse inverted confocal microscope equipped with an A1 imaging system, both controlled by the proprietary software NIS Elements v.5.0. Imaging was performed using a 60x (oil immersion, NA 1.4) objective lens. Dyes were excited in a sequential mode using the built-in laser lines: 403 nm (blue color), 488 nm (green color). Corresponding emissions were read in the following ranges: 425–475 nm (blue), 500–550 nm (green), using the manufacturer-provided filter sets. Images were acquired and analyzed using NIS Elements v.5.0. For E-cadherin, vimentin, SOX-2, and OCT-4 expression, the cells were observed using a fluorescence microscope Motic AE31 and images were acquired with a digital camera (Moticam 5.0 MP).

### Analyses of IL-8 and cytokine profile of primary BrC cultures

Levels of IL-8 were determined by an enzyme-linked immunosorbent assay (ELISA) using a sandwich ELISA kit (BD, Bioscience, San Jose, CA, USA, REF 555244) according to the manufacturer's instructions. To determine the cytokine profile of the different CMs, the concentration (in pgs/mL) of G-CSF, GM-CSF, IL-8, MCP-1, and metalloproteinases MMP-1 and MMP-2 were determined with the MILLIPLEX HCYTOMAG-60K Kit (EMD Millipore Corporation) following the manufacturer's recommended procedure. The analysis of data was performed in the xPONENT^®^ Software.

### Immunocytochemistry

BrC cells were stained with a rabbit monoclonal primary antibody anti-Estrogen Receptor (SP1, REF 790–4325), anti-Progesterone Receptor (1E2, REF 790–4296), and anti HER-2/neu (4B5, REF 790–2991) all from Ventana Medical Systems, Inc. (Ventana) and mouse monoclonal Androgen Receptor (F39.4.1, REF AM256-2ME) from Biogenex. Immunocytochemistry was performed using the UltraTek HRP Anti-Polyvalent Lab Pack from ScyTek Laboratories Inc. (REF UHP125), following the manufacturer's recommended procedure. Diaminobenzidine was used as the chromogen, and hematoxylin to counterstain. Images were acquired on a Zeiss Axioskop2Mot microscope through a Plan Neofluor 40× objective lens, using a color HVD-30 digital camera (Hitachi).

### Statistical analysis

The Prism software, version 5.01 (GraphPad) was used for statistical analysis using the unpaired Student's *t*-test. Data exhibiting a normal distribution were analyzed with the one-way analysis of variance (ANOVA) and the Tukey post hoc test in cases in which we had two or more groups of data, while for data lacking normality and/or homogeneity of variance the Kruskal-Wallis non-parametric test was used together with the Dunnett's post hoc test. Significant *P* values ≤ 0.05 are indicated by one asterisk ^*^, ≤ 0.01 by two asterisks ^**^ and ≤ 0.001 by three asterisks ^***^.

## SUPPLEMENTARY MATERIALS FIGURES AND TABLES







## Abbreviations

AR: Androgen receptor
BCSC: Breast cancer stem cell
BrC: Breast cancer
CDH1: E-cadherin
CM: Conditioned media
CSC: Cancer stem cell
CXCL12: C-X-C motif chemokine ligand 12
CXCR4: C-X-C chemokine receptor type 4
CXCR7: C-X-C chemokine receptor type 7
ECM: Extracellular matrix
EMT: Epithelial to mesenchymal transition
ER: Estrogen receptor
G-CSF: Granulocyte-colony-stimulating factor
GM-CSF: Granulocyte-macrophage colony-stimulating factor
HA-BrC: High aggressive breast cancer cell
HA-CMs: High aggressive conditioned media
HDAC3: Histone Deacetylase 3
IL-: Interleukin-
MCP-1: Monocyte chemoattractant protein-1
NA-BrC: Non-aggressive breast cancer cell
NA-CMs: Non-aggressive conditioned media
NFκB: Nuclear factor kappa-light-chain-enhancer of activated B cells
NIK: NFκB inducing kinase
PR: Progesterone receptor
STAT3: Signal transducer and activator of transcription 3
TGF-β: Transforming growth factor β
UBC: Ubiquitin

Genes differentially regulated with the arrays:

*ALCAM*: Activated leukocyte cell adhesion molecule
*ALDH1A1*: Aldehyde dehydrogenase 1 family: member A1
*ATXN1*: Ataxin 1
*CCL5*: C-C motif chemokine ligand 5
*CHEK1*: CHK1 checkpoint homolog (S; pombe)
*CTLA-4*: Cytotoxic T lymphocyte associated protein 4
*CXCR5*: C-X-C chemokine receptor type 5
*DACH1*: Dachshund homolog 1 (Drosophila)
*DLL1*: Delta-like 1 (Drosophila)
*EGF*: Epidermal growth factor
*ENG*: Endoglin
*FASL*: Fas ligand
*FGFR2*: Fibroblast growth factor receptor 2
*FOXP3*: Forkhead box P3
*GAPDH*: Glyceraldehyde-3-phosphate dehydrogenase
*GATA3*: GATA binding protein 3
*GSK3*β: Glycogen synthase kinase 3 beta
*GZMA:B*: Granzyme A: B
*HLA-*: Human leukocyte antigens
*ID1*: Inhibitor of DNA binding 1: dominant negative helix-loop-helix protein
*IFNG*: Interferon Gamma
*JAG1*: Jagged 1
*KITLG*: KIT ligand
*LIN28A*: Lin-28 homolog A (C; elegans)
*NOS2*: Nitric Oxide Synthase 2
*PDCD1*: Programmed Cell Death 1
*PIK3CA*: Phosphatidylinositol-4:5-bisphosphate 3-kinase catalytic subunit alpha
*PLAT*: Plasminogen activator: tissue
*PLAUR*: Plasminogen activator: urokinase receptor
*PROM1*: Prominin 1
*SAV1*: Salvador homolog 1 (Drosophila)
*SIRT1*: Sirtuin 1
*SPP1*: Secreted Phosphoprotein 1
*TLR*: Toll-like receptor
*ZEB*: Zinc Finger E-Box Binding Homeobox 1
