# Supplementary material for: An NFκB-dependent mechanism of tumor cell plasticity and lateral transmission of aggressive features
Source: Oncotarget. 2018 Jun 1;9(42):26679–700. doi: 10.18632/oncotarget.25465 (PMC6003573; doi:10.18632/oncotarget.25465)
Supplement: Supplementary file 1 [file oncotarget-09-26679-s001.pdf]

An NFκB-dependent mechanism of tumor cell plasticity and lateral transmission of aggressive features

SUPPLEMENTARY MATERIALS

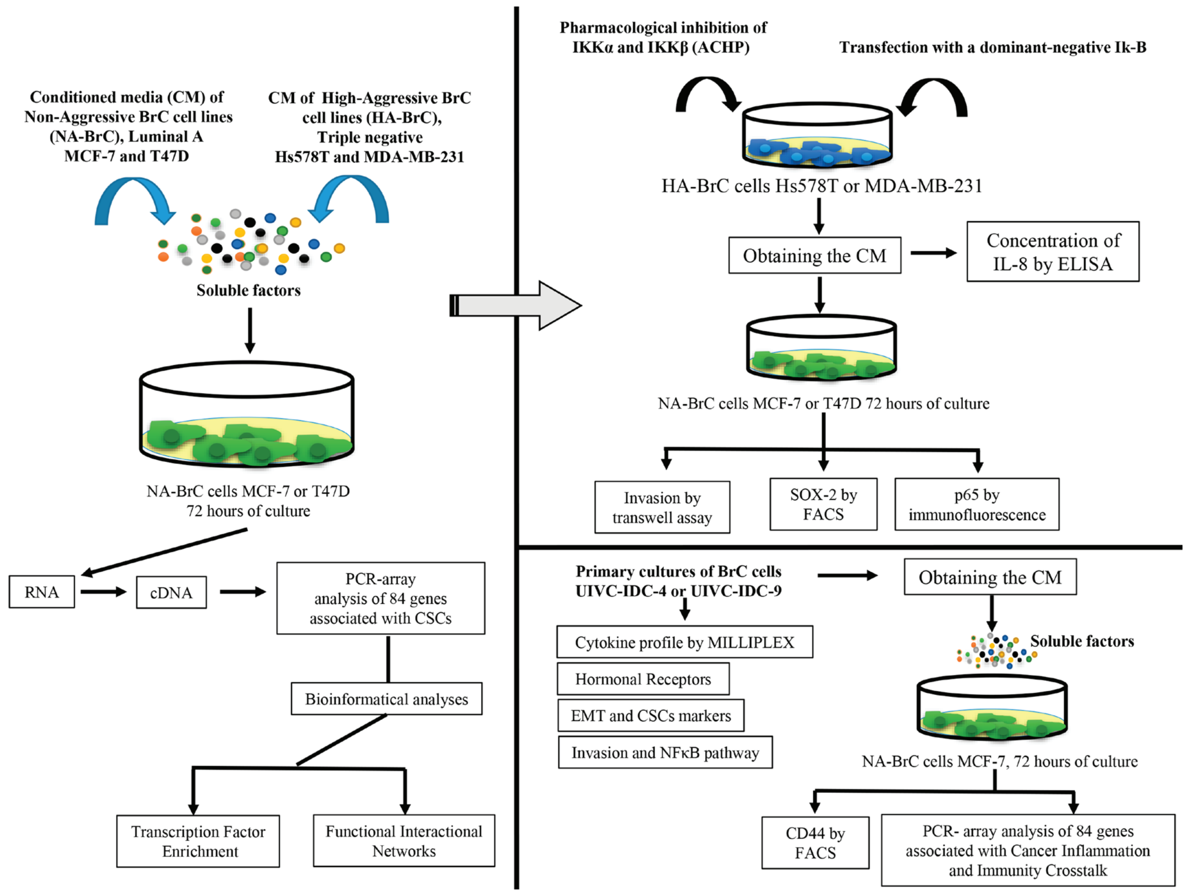

Supplementary Figure 1: Graphic representation of the experimental design.

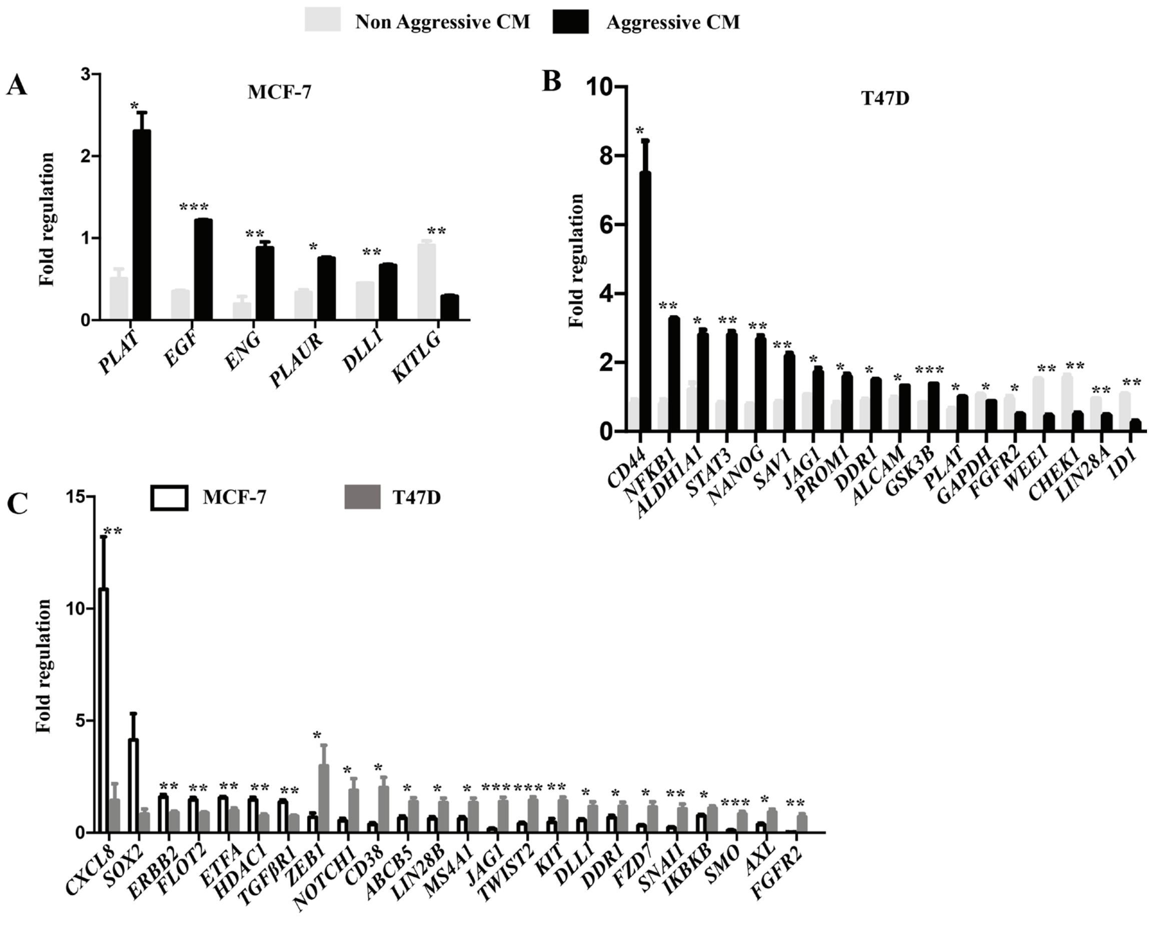

Supplementary Figure 2: Unsupervised unpaired student's *t*-test analyses of the genes found differentially expressed in NA-BrC cells. Analyses of the candidate genes differentially regulated after stimulation with NA- and HA-CMs in (**A**) MCF-7 and (**B**) T47D. (**C**) The genes found differentially expressed in the unstimulated (basal) conditions of MCF-7 and T47D cells.

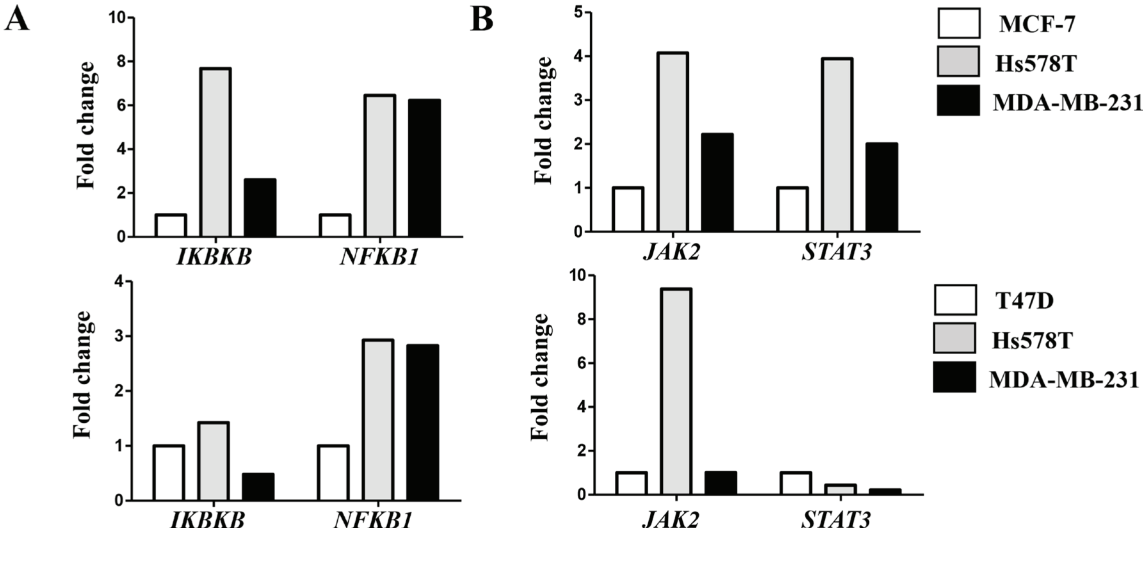

Supplementary Figure 3: Intrinsic expression signature of genes from the NFκB and JAK/STAT pathway in aggressive BrC cells and hormonal receptors. Basal levels of (**A**) *IKBKB* and *NFKB1* and (**B**) JAK2 and STAT3 found in the gene expression array. The graph shows the expression in HS78T and MDA-MB-231 cells normalized to HPRT1 and expressed as fold-difference from the normalized expression found in the MCF-7 (top) or T47D (bottom) cells.

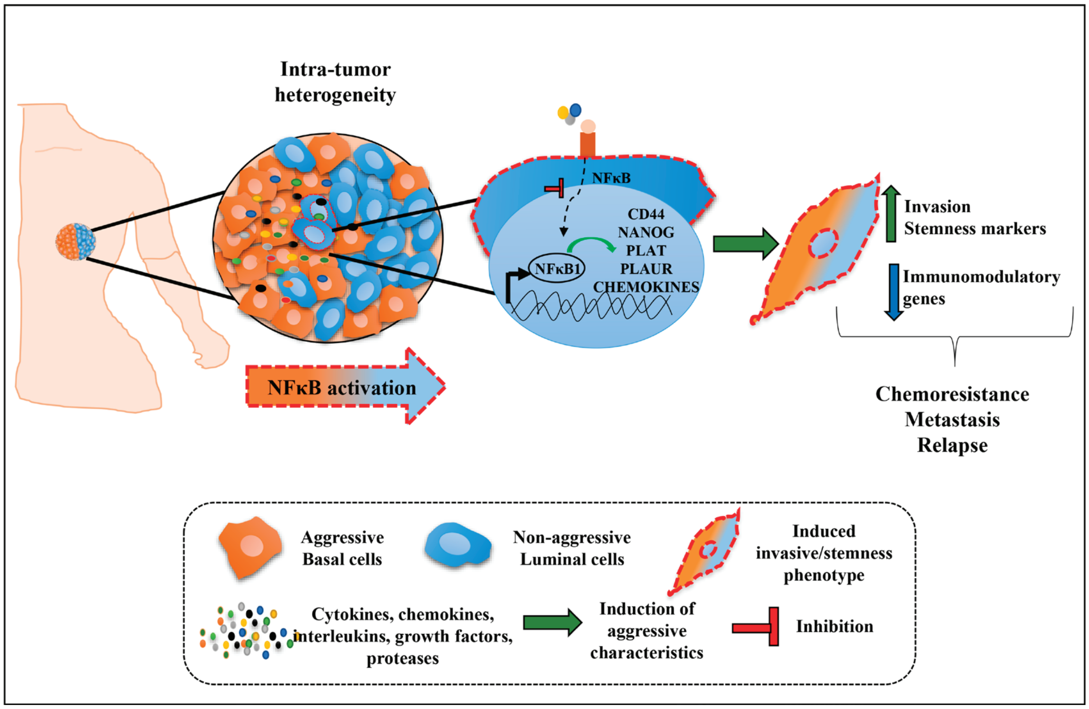

Supplementary Figure 4: Working model. BrC is a highly heterogeneous disease with high intra-tumoral diversity in which different molecular subtypes co-exist within the same tumor. We observed that highly aggressive basal tumor cells have an intrinsic inflammatory signature correlating with NFκB activation, and the capacity to laterally transmit aggressive cancer features into luminal tumor cells. Induced-aggressive cells are characterized either by the increased expression of the NFκB pathway or by the up-regulation of the NFκB target genes. These cells acquire a CSC-like phenotype, invasive properties and exhibited down-regulating of a set of immunomodulatory genes. The communication between different populations of BrC with different molecular subtypes may be critical to shape the tumor clinical response influencing chemoresistance, metastasis, and disease relapse.

Supplementary Table 1: Expression profiles of MCF-7 and T47D cells cultured with conditioned media from NA and HA-BrC cell lines. Expression of CSC-associated genes was quantified by a qRT-PCR array. Individual experiments were normalized against HPRT1 and fold expression was calculated normalizing against gene expression of the respective untreated MCF-7 or T47D cells. Downregulated genes are represented in blue (cutoff value ≤ −2, while upregulated genes are represented in red (cutoff value ≥ +2). *Duplicated experiments for which gene expression data were averaged after 2^DeltaCq calculation. Related to Supplementary Figure 1. See Supplementary_Table_1.

Supplementary Table 2: Signature of combined gene expression of MCF-7 and T47D cells induced after supervised cluster analysis

MCF7+T47 / NA-CM vs A-CM			
Unpaired 2-tailed *t*-test (R stats)			
STAT3	0.0025	DKK1	0.3302
GSK3B	0.0031	ITGA4	0.3330
NANOG	0.0032	ABCG2	0.3516
DACH1	0.0075	POU5F1	0.3569
NFKB1	0.0245	ENG	0.3587
CHEK1	0.0277	FLOT2	0.3681
NOTCH2	0.0284	FOXA2	0.3811
GATA3	0.0291	ID1	0.3980
WEE1	0.0298	FZD7	0.4299
ATXN1	0.0311	CXCL8	0.4596
PLAT	0.0331	FGFR2	0.4803
SIRT1	0.0432	BMI1	0.4897
CD24	0.0453	JAG1	0.4909
DNMT1	0.0587	ERBB2	0.5012
CD34	0.0676	LATS1	0.5022
THY1	0.0731	ALDH1A1	0.5309
KLF4	0.0754	ETFA	0.5539
MUC1	0.0872	KITLG	0.6055
DDR1	0.0882	ZEB2	0.6156
EGF	0.0909	TAZ	0.6331
ITGB1	0.1026	JAK2	0.6371
B2M	0.1109	MYCN	0.6375
KLF17	0.1155	ABCB5	0.6385
EPCAM	0.1486	ITGA6	0.6476
SAV1	0.1488	GAPDH	0.6612
ALCAM	0.1497	SOX2	0.6700
CD44	0.1541	TGFBR1	0.6924
PLAUR	0.1576	TWIST2	0.6948
DLL1	0.1680	SMO	0.7042
NOTCH1	0.1766	HDAC1	0.7159
LIN28A	0.1805	SNAI1	0.7210
ZEB1	0.1861	PTPRC	0.7225
PROM1	0.1896	MYC	0.7246
MERTK	0.2190	NOS2	0.7387
IKBKB	0.2251	ACTB	0.7397
PTCH1	0.2307	LIN28B	0.7762
TWIST1	0.2473	MS4A1	0.7762
WNT1	0.2539	KIT	0.8061
BMP7	0.2879	WWC1	0.8102
DLL4	0.2888	AXL	0.8158
CD38	0.3001	ATM	0.8241
PECAM1	0.3106	FOXP1	0.9055
MAML1	0.3157	YAP1	0.9708
ITGA2	0.3227		

Individual expression arrays were normalized against HPRT1 and fold expression was calculated normalizing against the respective MCF-7 or T47D basal expression. The values were averaged after the 2^DeltaCq calculation, applying the Student *t* test after stimulation with NA-CM and HA-CM. The genes with significant differential expression (p-value < 0.05) are highlighted in gray. Related to Figure 1.

Supplementary Table 3: ChiP-X transcription factor enrichment analysis

TF (PMID)	Target /Input	Targets/ Database	Fraction/ Input	Fraction/ Database	Difference	*P*-value	*Z*-score	Combined Score	Input CSC Genes
POU5F1 (Oct-4) (16153702)	4	622	0.3077	0.0207	0.2870	0.0001	−2.3553	21.3575	NANOG, STAT3, DACH1 & WEE1
NANOG (16153702)	5	1686	0.3846	0.0562	0.3284	0.0005	−2.4216	18.4265	GATA3, NANOG, WEE1, STAT3 & DACH1
MITF (21258399)	8	5578	0.6154	0.1859	0.4295	0.0008	−0.6334	4.5565	PLAT, SIRT1, STAT3, DACH1, WEE1, ATXN1, NFKB1 & GSK3B
SMAD3 (18955504)	5	1936	0.3846	0.0645	0.3201	0.0009	−1.4044	9.7983	GATA3, WEE1, ATXN1, NFKB1 & NOTCH2
SMAD2 (18955504)	5	1936	0.3846	0.0645	0.3201	0.0009	−1.4044	9.7983	GATA3, WEE1, ATXN1, NFKB1 & NOTCH2
STAT6 (20620947)	3	508	0.2308	0.0169	0.2138	0.0012	−2.0278	13.5789	STAT3, DACH1 & ATXN1
Sox-2 (16153702)	4	1278	0.3077	0.0426	0.2651	0.0017	−2.2538	14.3241	NANOG, STAT3, GSK3B & DACH1
AR (19668381)	6	3519	0.4615	0.1173	0.3443	0.0021	−1.0285	6.3185	PLAT, DACH1, WEE1, ATXN1, CD24 & GSK3B
RUNX1 (21571218)	7	5071	0.5385	0.1690	0.3695	0.0026	−0.1830	1.0881	PLAT, SIRT1, STAT3, WEE1, ATXN1, NFKB1 & NOTCH2
WT1 (19549856)	2	199	0.1538	0.0066	0.1472	0.0033	−1.8928	10.8063	STAT3 & NOTCH2

Potential transcription factors (TF) regulating the genes differentially expressed in NA-BrC cell lines cultured with the HA-CMs.

Supplementary Table 4: Expression profiles of MCF-7 cells cultured with the conditioned media from primary BrC cell lines derived from Mexican patients. Inflammation and Cancer Immunity Crosstalk-associated gene expression was quantified by qRT-PCR array. Individual experiments were normalized against HPRT1 and fold expression was calculated normalizing against the respective MCF-7 without CM. In blue are represented values for downregulated genes under a cutoff value ≤ −2, and in red upregulated genes above cutoff value ≥ +2. See Supplementary_Table_4.

Supplementary Table 5: Biological process associated with the down-regulated genes in MCF-7 cells cultured with CMs derived from primary cultures

Pathway description	Observed gene count	False discovery rate	Matching proteins in your network (labels)
Immune response	19	2.08E-13	AICDA,BCL2,CCL18,CCL2,CCR10,CCR7,CD274,CSF1,CSF2,CTLA4,CXCL12,CXCL9,FASLG,GZMA,HLA-A,HLA-C,IL17A,PDCD1,TLR4
Cell surface receptor signaling pathway	21	5.94E-13	BCL2,CCL2,CCR10,CCR7,CD274,CSF1,CTLA4,CXCL12,CXCL9,CXCR1,CXCR2,CXCR3,FASLG,HLA-A,HLA-B,HLA-C,IFNG,IL12B,IL2,MYC,TLR4
Cytokine-mediated signaling pathway	13	1.76E-12	CCL2,CCR10,CCR7,CSF1,CXCL12,CXCL9,CXCR1,CXCR2,HLA-A,HLA-B,HLA-C,IFNG,IL12B
Response to cytokine	14	2.15E-11	BCL2,CCL2,CCR10,CCR7,CSF1,CXCL12,CXCL9,CXCR1,CXCR2,HLA-A,HLA-B,HLA-C,IL12B,IL17A
Negative regulation of immune system process	12	2.51E-11	CCL2,CD274,CTLA4,CXCL12,HLA-A,HLA-B,IDO1,IL12B,IL2,MYC,PDCD1,TLR4
Regulation of immune system process	17	4.20E-11	BCL2,CCL2,CCR7,CD274,CSF1,CTLA4,CXCL12,CXCL9,CXCR3,HLA-A,HLA-B,HLA-C,IDO1,IFNG,IL12B,IL17A,MYC
Cellular response to cytokine stimulus	13	4.20E-11	CCL2,CCR10,CCR7,CSF1,CXCL12,CXCL9,CXCR1,CXCR2,HLA-A,HLA-B,HLA-C,IL12B,IL17A
Chemokine-mediated signaling pathway	7	1.50E-10	CCL2,CCR10,CCR7,CXCL12,CXCL9,CXCR1,CXCR2
Positive regulation of cell proliferation	14	2.27E-10	BCL2,CCL2,CD274,CSF1,CSF2,CXCL12,CXCR2,CXCR3,FASLG,IFNG,IL12B,IL2,MYC,TLR4
Cellular response to organic substance	18	2.27E-10	AICDA,BCL2,CCL2,CCR10,CCR7,CSF1,CSF2,CXCL12,CXCL9,CXCR1,CXCR2,HLA-A,HLA-B,HLA-C,IL12B,IL17A,MYC,TLR4
Cellular response to chemical stimulus	19	4.27E-10	AICDA,BCL2,CCL18,CCL2,CCR10,CCR7,CSF1,CSF2,CXCL12,CXCL9,CXCR1,CXCR2,HLA-A,HLA-B,HLA-C,IL12B,IL17A,MYC,TLR4
Cell chemotaxis	9	5.14E-10	CCL18,CCL2,CCR7,CXCL12,CXCL9,CXCR1,CXCR2,CXCR3,IFNG
Immune system process	18	5.48E-10	CCL18,CCL2,CCR10,CCR7,CD274,CSF1,CSF2,CTLA4,CXCR1,CXCR2,CXCR3,FASLG,GZMA,HLA-A,HLA-C,IL17A,PDCD1,TLR4
Regulation of multicellular organismal process	19	5.48E-10	BCL2,CCL2,CCR7,CD274,CSF1,CSF2,CTLA4,CXCL12,CXCR3,FASLG,HLA-B,IDO1,IFNG,IL12B,IL17A,IL2,MYC,PDCD1,TLR4
Response to lipopolysaccharide	10	9.85E-10	AICDA,CCL2,CCR7,CSF2,CXCL9,FASLG,IDO1,IFNG,IL12B,TLR4
Response to organic substance	19	1.10E-09	AICDA,CCL2,CCR10,CCR7,CSF1,CSF2,CXCL12,CXCL9,CXCR1,CXCR2,FASLG,HLA-A,HLA-B,HLA-C,IDO1,IL12B,IL17A,MYC,TLR4
Positive regulation of cell death	12	2.14E-09	BCL2,CTLA4,CXCR3,FASLG,GZMA,IDO1,IFNG,IL12B,IL17A,MYC,PDCD1,TLR4
Regulation of multicellular organismal development	16	2.14E-09	BCL2,CCL2,CSF1,CTLA4,CXCL12,CXCR3,FASLG,HLA-B,IDO1,IFNG,IL12B,IL17A,IL2,MYC,PDCD1,TLR4
Positive regulation of immune system process	13	3.52E-09	BCL2,CCL2,CCR7,CD274,CSF1,CTLA4,CXCL12,CXCL9,IDO1,IFNG,IL12B,IL17A,PDCD1
Positive regulation of lymphocyte activation	9	4.26E-09	BCL2,CCL2,CCR7,CD274,CTLA4,IFNG,IL12B,PDCD1,TLR4

The program STRING was used for this analysis.

Supplementary Table 6: Biological process associated to up-regulated genes in MCF-7 cultured with CMs derived from primary cultures

Pathway description	Observed gene count	False discovery rate	Matching proteins in your network
Chemokine-mediated signaling pathway	7	3.27E-11	CCL5,CCR1,CCR2,CXCL10,CXCR4,CXCR5,CXCR7
Immune response	14	9.30E-11	CCL22,CCL28,CCL5,CCR1,CCR2,CCR9,CXCL10,CXCR5,EGF,GBP1,IL15,NOS2,TLR3,TNFSF10
Cellular response to cytokine stimulus	10	1.02E-08	CCL5,CCR1,CCR2,CXCL10,CXCR4,CXCR5,CXCR7,GBP1,NOS2,STAT3
Cytokine-mediated signaling pathway	9	2.19E-08	CCL5,CCR1,CCR2,CXCL10,CXCR4,CXCR5,CXCR7,GBP1,STAT3
Chemotaxis	10	2.50E-08	CCL22,CCL28,CCL5,CCR1,CCR2,CCR9,CXCL10,CXCR4,CXCR5,CXCR7
Inflammatory response	9	2.80E-08	CCL22,CCR1,CCR2,CXCL10,CXCR4,IL15,NOS2,STAT3,TLR3
Cellular response to chemical stimulus	14	3.71E-08	CCL22,CCL28,CCL5,CCR1,CCR2,CXCL10,CXCR4,CXCR5,CXCR7,EGF,GBP1,IL15,NOS2,STAT3
Cell chemotaxis	7	5.60E-08	CCL22,CCL28,CCL5,CCR1,CCR2,CXCL10,CXCR4
Response to external stimulus	13	6.56E-08	CCL22,CCL28,CCL5,CCR1,CCR2,CCR9,CXCR4,CXCR5,CXCR7,GBP1,IL15,NOS2,TLR3
Immune system process	13	1.21E-07	CCL22,CCL28,CCL5,CCR1,CCR2,CCR9,CXCR4,CXCR5,EGF,GBP1,NOS2,TLR3,TNFSF10
Positive regulation of monocyte chemotaxis	4	3.17E-07	CCL5,CCR1,CCR2,CXCL10
dendritic cell chemotaxis	4	7.47E-07	CCL5,CCR1,CCR2,CXCR4
Regulation of immune system process	11	7.60E-07	CCL28,CCL5,CCR1,CCR2,CXCL10,CXCR7,EGF,GBP1,IL15,NOS2,TLR3
Defense response	11	9.96E-07	CCL22,CCR1,CCR2,CCR9,CXCR4,EGF,GBP1,IL15,NOS2,STAT3,TLR3
Cell surface receptor signaling pathway	12	2.63E-06	CCL5,CCR1,CCR2,CXCL10,CXCR4,CXCR5,CXCR7,EGF,GBP1,STAT3,TLR3,TNFSF10
Response to chemical	14	1.03E-05	CCL22,CCL28,CCL5,CCR1,CCR2,CXCR4,CXCR5,CXCR7,EGF,GBP1,IL15,NOS2,STAT3,TLR3
Leukocyte chemotaxis	5	1.05E-05	CCL5,CCR1,CCR2,CXCL10,CXCR4
Cellular response to organic substance	11	1.27E-05	CCR1,CCR2,CXCL10,CXCR4,CXCR5,CXCR7,EGF,GBP1,IL15,NOS2,STAT3
Response to virus	6	1.38E-05	CCL22,CCL5,CXCL10,CXCR4,GBP1,TLR3
Regulation of phosphate metabolic process	10	2.71E-05	CCL5,CCR1,CCR2,CXCL10,CXCR4,CXCR7,GBP1,IL15,NOS2,STAT3

The program STRING was used for this analysis.
