# Supplementary material for: An NFκB-dependent mechanism of tumor cell plasticity and lateral transmission of aggressive features
Source: Oncotarget. 2018 Jun 1;9(42):26679–700. doi: 10.18632/oncotarget.25465 (PMC6003573; doi:10.18632/oncotarget.25465)
Supplement: Supplementary file 2 [file oncotarget-09-26679-s002.docx]

**Supplementary Table 1: Expression profiles of MCF-7 and T47D cells cultured with conditioned media from NA and HA-BrC cell lines**

|  | **Normalized to untreated MCF-7** | | | | **Normalized to untreated T47D** | | | |
| --- | --- | --- | --- | --- | --- | --- | --- | --- |
| **Gene Symbol** | MCF7 + MCF7 CM | MCF7 + T47D CM | MCF7 + HS578T CM * | MCF7 + MDA-MB-231 CM * | T47D + MCF7 CM | T47D + T47D CM | T47D + HS578T CM * | T47D + MDA-MB-231 CM * |
| ABCB5 | -1.31 | -2.77 | -1.51 | -1.73 | 1.06 | 1.52 | 1.00 | -0.57 |
| ABCG2 | -1.56 | -1.13 | 1.35 | -1.44 | -1.28 | 1.03 | -1.26 | -1.21 |
| ALCAM | -1.04 | -1.23 | 1.10 | -1.08 | -1.18 | -1.01 | 1.25 | -0.66 |
| ALDH1A1 | 2.39 | -2.20 | -1.38 | -1.73 | -1.01 | 1.41 | 2.67 | -0.36 |
| ATM | 1.39 | 1.16 | 1.44 | -1.04 | -1.06 | 1.39 | 1.56 | 1.69 |
| ATXN1 | 1.05 | 1.08 | 1.38 | 1.47 | 1.01 | 1.01 | 2.25 | 2.06 |
| AXL | -2.45 | -4.76 | -3.17 | -2.37 | 1.01 | 1.06 | -1.04 | -1.12 |
| BMI1 | 1.21 | -1.01 | 1.28 | -1.46 | -1.07 | -1.01 | 1.01 | -1.21 |
| BMP7 | -1.58 | -1.40 | -1.32 | -2.78 | -1.01 | -1.09 | 3.27 | 1.91 |
| CD24 | -2.46 | -1.07 | 1.23 | 1.70 | -1.18 | 1.26 | 3.19 | 2.33 |
| CD34 | -1.31 | -2.77 | -1.65 | -1.73 | -2.68 | -1.88 | -1.16 | -1.53 |
| CD38 | -3.78 | -3.63 | -2.18 | -2.38 | 1.06 | 1.52 | 2.22 | 3.32 |
| CD44 | 1.39 | 1.27 | 1.57 | 1.81 | -1.09 | -1.39 | 5.92 | 9.20 |
| CHEK1 | 1.40 | 1.16 | 1.18 | -1.10 | 1.47 | 1.64 | -2.01 | -2.06 |
| DACH1 | 1.06 | -1.25 | -1.56 | -4.11 | -1.01 | -1.16 | -1.46 | -1.72 |
| DDR1 | -2.30 | -1.60 | -1.41 | -1.40 | -1.06 | -1.22 | 1.37 | 1.39 |
| DKK1 | -1.78 | -1.20 | -1.08 | -1.58 | 2.11 | 1.23 | 1.11 | -1.03 |
| DLL1 | -2.20 | -2.22 | -1.60 | -1.78 | -1.05 | -1.39 | 1.73 | 1.85 |
| DLL4 | -4.08 | -2.62 | -3.27 | -1.14 | 1.41 | 1.17 | 1.71 | 8.17 |
| DNMT1 | 1.25 | 1.26 | 1.03 | -1.94 | 1.02 | -1.25 | -2.47 | -2.86 |
| EGF | -2.95 | -2.75 | 1.17 | 1.13 | -1.05 | -1.31 | 1.65 | 4.72 |
| ENG | -9.78 | -3.46 | -1.08 | 1.26 | -1.04 | -1.25 | -1.21 | -1.98 |
| EPCAM | -1.09 | -1.04 | 1.23 | -1.01 | 1.06 | 1.01 | 1.17 | 1.09 |
| ERBB2 | 1.61 | 1.93 | 1.35 | 1.44 | -1.03 | -1.13 | -1.09 | -1.16 |
| ETFA | 1.60 | 1.47 | 1.57 | 1.38 | 1.05 | 1.28 | -1.18 | -1.15 |
| FGFR2 | -50.21 | -29.86 | -17.63 | -34.36 | 1.02 | -1.16 | -2.21 | -1.87 |
| FLOT2 | 1.25 | 1.30 | 1.60 | 1.47 | -1.16 | -1.22 | -1.09 | -1.08 |
| FOXA2 | -3.18 | -4.86 | -2.49 | -2.25 | 1.06 | 29.65 | -1.13 | 1.75 |
| FOXP1 | 1.51 | 1.77 | 1.31 | -1.11 | -1.04 | -1.26 | 1.48 | 1.27 |
| FZD7 | -4.72 | -3.41 | -2.64 | -3.65 | -1.08 | -1.16 | -1.06 | 1.74 |
| GATA3 | -1.53 | 1.25 | -1.91 | -3.57 | -1.16 | -1.19 | -1.49 | -2.31 |
| GSK3B | -1.56 | -1.08 | 1.05 | 1.06 | -1.19 | -1.21 | 1.34 | 1.41 |
| HDAC1 | 1.37 | 1.56 | 1.60 | 1.11 | -1.15 | -1.11 | -1.55 | -1.68 |
| ID1 | -1.84 | -2.77 | 1.06 | -1.74 | 1.04 | 1.09 | -2.86 | -3.44 |
| IKBKB | -1.27 | -1.58 | -1.20 | -1.51 | 1.00 | -1.17 | 1.16 | 1.38 |
| CXCL8 | 5.94 | 10.34 | 9.33 | 32.61 | -1.40 | -1.28 | 4.44 | 1.25 |
| ITGA2 | 1.48 | 1.79 | 1.33 | -1.96 | -1.21 | -1.21 | 1.45 | 1.16 |
| ITGA4 | -1.31 | -2.77 | 1.68 | -1.73 | 1.06 | 1.52 | -1.13 | 1.75 |
| ITGA6 | 2.57 | -1.53 | 1.57 | -0.51 | -1.06 | 1.00 | 1.49 | 1.27 |
| ITGB1 | -1.21 | -1.03 | 1.43 | -0.90 | -1.10 | 1.21 | 1.31 | 1.11 |
| JAG1 | -15.67 | -6.41 | -5.40 | -4.64 | 1.07 | 1.05 | 1.67 | 1.55 |
| JAK2 | -1.05 | 1.40 | -2.68 | -4.37 | -1.03 | 1.09 | 1.56 | 4.20 |
| KIT | -1.87 | -1.11 | -4.43 | -6.72 | 1.06 | 1.52 | 1.21 | 1.75 |
| KITLG | -1.04 | -1.16 | -3.32 | -3.37 | 1.13 | -1.06 | 4.29 | 2.23 |
| KLF17 | -1.29 | 1.39 | 1.25 | 1.09 | -1.21 | -4.63 | 1.76 | 1.55 |
| KLF4 | -2.13 | -1.48 | 1.41 | 1.35 | -1.07 | -1.25 | 2.68 | 4.29 |
| LATS1 | -1.24 | 1.33 | 1.13 | -1.02 | -1.26 | -1.27 | 1.68 | 1.42 |
| LIN28A | -2.57 | -1.60 | -1.69 | -1.78 | -1.05 | -1.06 | -2.30 | -1.75 |
| LIN28B | -1.31 | -2.77 | -1.95 | -1.73 | 1.06 | 1.52 | -1.13 | 1.75 |
| MAML1 | -1.17 | -1.20 | -1.05 | -1.39 | -1.21 | -1.40 | -1.24 | -1.20 |
| MERTK | -1.12 | -2.95 | 1.02 | -1.70 | 1.23 | -1.55 | 1.41 | 2.00 |
| MS4A1 | -1.31 | -2.77 | -1.95 | -1.73 | 1.06 | 1.52 | -1.13 | 1.75 |
| MUC1 | 1.28 | 1.51 | 2.67 | 6.14 | 1.00 | 0.76 | 2.15 | 1.84 |
| MYC | 1.34 | 1.23 | -1.04 | -1.52 | -1.04 | -1.21 | 1.75 | 1.02 |
| MYCN | -7.89 | -2.58 | -2.47 | 2.39 | 1.26 | -1.71 | -2.99 | 1.05 |
| NANOG | -1.68 | 1.34 | 1.92 | 3.22 | -1.39 | -1.24 | 5.48 | 6.90 |
| NFKB1 | 1.23 | 1.35 | 1.90 | 1.91 | -1.09 | -1.54 | 3.03 | 3.20 |
| NOS2 | -2.77 | -1.38 | -1.15 | 1.39 | -1.61 | -1.80 | -1.53 | -3.86 |
| NOTCH1 | -3.78 | -1.95 | -1.54 | -1.86 | 1.19 | -1.11 | 2.23 | 3.11 |
| NOTCH2 | 1.16 | -1.29 | -1.58 | -2.07 | 1.54 | -1.20 | -2.61 | 1.02 |
| PECAM1 | 8.51 | 11.79 | 12.07 | 29.67 | -5.46 | -3.84 | -1.77 | -3.32 |
| PLAT | -1.60 | -2.58 | 2.26 | 2.99 | -1.48 | -1.78 | 1.15 | 1.05 |
| PLAUR | -3.32 | -2.69 | -1.04 | -1.73 | -1.08 | -1.84 | -1.62 | 2.83 |
| PO5F1 | -1.36 | -1.65 | -1.26 | -1.91 | 1.38 | -1.49 | 1.28 | 3.81 |
| PROM1 | -2.04 | 1.14 | 1.91 | 39.14 | -1.18 | -1.58 | 1.81 | 4.03 |
| PTCH1 | -1.88 | -1.25 | -1.02 | -2.10 | -1.78 | -1.56 | -1.51 | 1.41 |
| PTPRC | 46.21 | 13.27 | 12.28 | 13.82 | 1.06 | 1.52 | -1.13 | 2.01 |
| SAV1 | -1.06 | -1.09 | 1.05 | -1.25 | -1.15 | -1.29 | 1.96 | 2.42 |
| SIRT1 | 1.17 | 1.35 | 1.60 | 1.35 | -1.05 | -1.18 | 1.71 | 2.67 |
| SMO | -7.52 | -15.14 | -14.31 | -7.77 | 1.17 | -1.29 | -1.62 | -1.24 |
| SNAI1 | -6.02 | -6.06 | -3.77 | -2.57 | 1.12 | -1.20 | -1.56 | 2.00 |
| SOX2 | 3.53 | 3.20 | 13.11 | 7.76 | 1.00 | -1.54 | 1.24 | 1.60 |
| STAT3 | -1.19 | -1.17 | 1.51 | 2.44 | -1.18 | -1.36 | 2.44 | 3.27 |
| TAZ | -1.29 | -1.47 | -1.66 | -2.97 | -1.33 | -1.53 | -1.45 | -1.20 |
| TGFBR1 | 1.33 | 1.60 | 1.32 | 1.10 | -1.34 | -1.34 | -1.13 | -1.33 |
| THY1 | -2.08 | -1.84 | -2.88 | -2.75 | -1.28 | -2.46 | -4.47 | -10.63 |
| TWIST1 | -1.31 | -2.77 | -1.95 | -1.73 | -1.16 | -1.53 | 1.06 | 1.87 |
| TWIST2 | -2.00 | -4.23 | -2.97 | -2.64 | 1.06 | 1.52 | 1.29 | 1.75 |
| WEE1 | 1.72 | -1.06 | 1.38 | -1.33 | 1.54 | 1.46 | -2.31 | -1.48 |
| WNT1 | -1.31 | -2.60 | -1.95 | -1.73 | -1.08 | -1.83 | -4.60 | -7.03 |
| WWC1 | -1.55 | -1.04 | -1.38 | -1.53 | -1.17 | -1.60 | -1.32 | 1.09 |
| YAP1 | 1.04 | 1.04 | 1.25 | -1.55 | -1.19 | -1.18 | -1.29 | -1.23 |
| ZEB1 | -4.38 | -1.65 | -1.36 | 1.00 | 1.62 | 1.52 | 4.18 | 5.19 |
| ZEB2 | 2.31 | -2.77 | 1.34 | 1.37 | -6.73 | -1.02 | -1.12 | 2.28 |
| ACTB | -1.05 | -1.11 | 1.10 | -1.23 | -1.08 | -1.21 | -1.53 | -2.05 |
| B2M | -2.00 | -1.60 | -1.42 | 1.62 | -1.09 | -1.06 | 2.39 | 5.46 |
| GAPDH | -1.37 | -1.34 | 1.27 | -1.63 | 1.09 | 1.02 | -1.27 | -1.19 |

Expression of CSC-associated genes was quantified by a qRT-PCR array. Individual experiments were normalized against HPRT1 and fold expression was calculated normalizing against gene expression of the respective untreated MCF-7 or T47D cells. Downregulated genes are represented in blue (cutoff value ≤ -2, while upregulated genes are represented in red (cutoff value ≥ +2). * Duplicated experiments for which gene expression data were averaged after 2^DeltaCq calculation. Related to Supplementary Figure 1.
