# Supplementary material for: An NFκB-dependent mechanism of tumor cell plasticity and lateral transmission of aggressive features
Source: Oncotarget. 2018 Jun 1;9(42):26679–700. doi: 10.18632/oncotarget.25465 (PMC6003573; doi:10.18632/oncotarget.25465)
Supplement: Supplementary file 3 [file oncotarget-09-26679-s003.docx]

**­ Supplementary Table 4: Expression profiles of MCF-7 cells cultured with the conditioned media from primary BrC cell lines derived from Mexican patients. ­­­­**

|  | **Normalized to untreated MCF-7** | |
| --- | --- | --- |
| **Gene Symbol** | MCF7 + UIVC-ICD4 | MCF7 + UIVC-ICD9 |
| ACKR3 | 26.5382 | 8.8766 |
| AICDA | -3.5308 | -2.9282 |
| BCL2 | -3.0738 | -3.9449 |
| BCL2L1 | 1.007 | -1.4845 |
| CCL18 | -3.5308 | -2.9282 |
| CCL2 | -206.5003 | -14.42 |
| CCL20 | -1.0353 | 1.1408 |
| CCL21 | -1.1975 | 1.1408 |
| CCL22 | 9.3179 | 11.4716 |
| CCL28 | 6.5887 | 3.8906 |
| CCL4 | -1.057 | 1.3287 |
| CCL5 | 9.1896 | 8.5742 |
| CCR1 | 47.5048 | 44.6318 |
| CCR10 | -3.7321 | -2.0849 |
| CCR2 | 2.4623 | 2.6574 |
| CCR4 | -1.2658 | -1.8025 |
| CCR7 | -7.7812 | -4.084 |
| CCR9 | 3.2716 | 2.3784 |
| CD274 | -7.0128 | -3.4343 |
| CSF1 | -2.8879 | -2.3134 |
| CSF2 | -3.5308 | -2.9282 |
| CSF3 | 5.6962 | -2.639 |
| CTLA4 | -3.5308 | -2.9282 |
| CXCL1 | 1.9319 | 1.6358 |
| CXCL10 | 3.4105 | 3.0525 |
| CXCL11 | 1.2483 | -2.9282 |
| CXCL12 | -3.0105 | -2.5669 |
| CXCL2 | 1 | -1.3287 |
| CXCL5 | -1.3195 | -4.1699 |
| CXCL9 | -3.5308 | -2.9282 |
| CXCR1 | -3.5308 | -2.7511 |
| CXCR2 | -2.3295 | -9.4479 |
| CXCR3 | -3.5308 | -2.9282 |
| CXCR4 | 8.6939 | 11.3137 |
| CXCR5 | 3.6808 | 5.7757 |
| EGF | 9.6465 | 10.1261 |
| EGFR | 1.7053 | 1.3947 |
| FASLG | -3.5308 | -2.9282 |
| FOXP3 | -1.4641 | -2.2815 |
| GBP1 | 8.3397 | 9.3827 |
| GZMA | -3.5308 | -2.9282 |
| GZMB | -3.4343 | -1.3472 |
| HIF1A | 1.6133 | 1.4948 |
| HLA-A | -2.9897 | -3.5554 |
| HLA-B | -3.5308 | -2.9282 |
| HLA-C | -3.5308 | -2.9282 |
| IDO1 | -3.5308 | -2.9282 |
| IFNG | -3.5308 | -2.9282 |
| IGF1 | -3.3404 | -1.1173 |
| IL10 | -1.9588 | -2.9282 |
| IL12A | 1 | 2.3134 |
| IL12B | -3.5308 | -2.9282 |
| IL13 | -1.9185 | -4.4076 |
| IL15 | 4.5002 | 11.7942 |
| IL17A | -3.5308 | -2.9282 |
| IL1A | 2.4623 | 2.9282 |
| IL1B | -1.057 | 1.1251 |
| IL2 | -3.249 | -2.9282 |
| IL23A | 1.6586 | 1.6245 |
| IL4 | -1.2397 | -1.0353 |
| IL6 | 1.6472 | -2.4794 |
| CXCL8 | 1.6586 | 1.5263 |
| IRF1 | 1.9725 | 3.8106 |
| KITLG | 1.3379 | -1.1096 |
| MICA | 3.4822 | 3.8371 |
| MICB | 1.9319 | 1.2397 |
| MIF | 1.1096 | 1.257 |
| MYC | -3.9177 | -3.5554 |
| MYD88 | 1.9588 | 1.7777 |
| NFKB1 | -1.6133 | -1.4044 |
| NOS2 | 7.1602 | 5.3889 |
| PDCD1 | -3.3404 | -2.9282 |
| PTGS2 | -1.2142 | 1.4241 |
| SPP1 | -1.0718 | -2.9282 |
| STAT1 | 2.2501 | 1.5583 |
| STAT3 | 4.3169 | 3.8371 |
| TGFB1 | -1.3755 | -1.057 |
| TLR2 | -3.5308 | -1.4948 |
| TLR3 | 2.0139 | 2.514 |
| TLR4 | -3.5308 | -2.9282 |
| TNF | -9.5137 | -1.9862 |
| TNFSF10 | 9.3827 | 19.5622 |
| TP53 | -1.879 | -1.9453 |
| VEGFA | -1.3851 | -1.5369 |
| ACTB | -1.007 | -1.8404 |
| B2M | 2.0562 | 1.6133 |
| GAPDH | -1.7777 | -1.9725 |

Inflammation and Cancer Immunity Crosstalk-associated gene expression was quantified by qRT-PCR array. Individual experiments were normalized against HPRT1 and fold expression was calculated normalizing against the respective MCF-7 without CM. In blue are represented values for downregulated genes under a cutoff value ≤ -2, and in red upregulated genes above cutoff value ≥ +2.
